# Cabbage Detection and Counting Using YOLOv8-CBAM and Improved BoT-SORT for Greenhouse and Open Field Cultivation in Korea

**DOI:** 10.3390/s26196268

**Published:** 2026-10-02

**Authors:** Md Nasim Reza, Md Razob Ali, Md Mamunur Rashid, Hee-Bok Lee, Young Kyun Jang, Ezatullah Zakir, Cholet Nyangoma Atulinda, Sun-Ok Chung

**Affiliations:** 1 Department of Agricultural Machinery Engineering, Graduate School, Chungnam National University, Daejeon 34134, Republic of Korea; reza5575@cnu.ac.kr (M.N.R.); ali1999@o.cnu.ac.kr (M.R.A.); zak14@o.cnu.ac.kr (E.Z.); choletatulinda2@o.cnu.ac.kr (C.N.A.); 2 Department of Smart Agricultural Systems, Graduate School, Chungnam National University, Daejeon 34134, Republic of Korea; mamun@o.cnu.ac.kr; 3 Agricultural Corporation Cheongmyeong Co., Ltd., Buyeo 33202, Republic of Korea; chfarm2001@hanmail.net; 4 GreenCS Co., Ltd., Gwangju 57309, Republic of Korea; green_jyk@naver.com

**Keywords:** precision agriculture, cabbage seedling, plant counting, BoT-SORT, computer vision

## Abstract

Accurate plant counting supports stand assessment and crop management, but canopy overlap, camera motion, and temporary occlusion complicate video-based monitoring. This study developed an RGB image- and video-based framework for detecting, tracking, and counting cabbage seedlings and grown plants under greenhouse and open-field conditions. The framework integrated YOLOv8n with convolutional block attention modules (CBAM) and an improved BoT-SORT procedure incorporating trajectory persistence, short-gap relinking, class-consistent identity management, and counting-event filtering. Detector performance was evaluated across five independent training runs, counting performance across nine held-out videos, and tracking performance on four manually annotated videos. YOLOv8n-CBAM achieved mean precision, recall, mAP@0.50, and mAP@0.50:0.95 values of 0.880 ± 0.007, 0.920 ± 0.007, 0.960 ± 0.006, and 0.600 ± 0.008, respectively (mean ± sample standard deviation). The complete framework achieved 99.27% mean counting accuracy, a mean absolute error of 0.56 plants, a root mean square error of 0.88 plants, and an R^2^ of 0.997. On the annotated subset, Improved BoT-SORT increased mean IDF1 from 79.6% to 82.3% and mean HOTA from 74.3% to 83.2%, while reducing total identity switches from 23 to 12. Complete-pipeline throughput was 23.38 frames per second on an RTX 3090 workstation. These findings support cabbage population monitoring under the evaluated conditions; independent-site validation, tracking-component ablation, and deployment-device testing remain necessary to establish broader applicability.

## 1. Introduction

Cabbage (*Brassica rapa* L. ssp. Pekinensis) is one of the most significant crops in the Republic of Korea, serving as a fundamental ingredient in kimchi, a traditional and widely consumed Korean dish [1,2]. The Republic of Korea ranked as the fourth-largest producer of Brassica cabbages in the world, with an annual production of 2,555,876 tons [1,3]. As the demand for high-quality cabbage production grows, precision agriculture techniques are increasingly being adopted to enhance yield estimation, optimize farm management, and improve resource utilization [4]. Among these techniques, automated seedling counting during post-transplantation and early growth stages is highly beneficial for monitoring and evaluation seedling development, establishment, detecting seedling gaps, ensure spatial uniformity, and supporting timely management works, thereby improving crop monitoring efficiency and decision-making. However, traditional methods of seedling counting, which rely on manual labor or simple image processing techniques, are time-consuming, prone to human error, and inefficient for large-scale agricultural operations [5,6].

Precision agriculture has revolutionized modern farming by introducing advanced technologies that enhance productivity, sustainability, and operational efficiency [7]. One of the key components of precision agriculture is crop monitoring, which involves the systematic collection and analysis of data throughout different growth stages. Early-stage monitoring, particularly after transplantation, is essential for evaluating seedling survival rates, detecting growth anomalies, and optimizing resource allocation [8]. Accurate seedling counting enables farmers to make informed decisions regarding fertilization, irrigation, pest control, and replanting, thereby improving overall crop health and yield [9]. In vegetable production, where individual plants often have relatively high economic value and management is strongly influenced by plant spacing and stand uniformity, rapid determination of the actual crop population can be particularly important [6,8,10]. Furthermore, continuous crop counting and monitoring during the growing period can provide valuable information on plant density, crop vigor, stress, and spatial variability, while supporting pre-harvest yield estimation and improved harvest planning [11,12].

Traditional methods for counting cabbage seedlings involve manual inspections or simple image analysis techniques, both of which present significant challenges. Manual counting is labor-intensive, time-consuming, and impractical for large-scale farms. Image analysis methods, while offering some automation, often struggle with varying lighting conditions, occlusions, and the complex background environments found in open fields [6,10]. Additionally, existing automated systems may require expensive hardware, making them inaccessible for many farmers [10,13,14]. Therefore, a reliable, cost-effective, and scalable solution is needed to streamline seedling counting and crop monitoring under real-world agricultural conditions.

Computer vision and deep learning provide an increasingly practical means of addressing these limitations. Deep convolutional neural networks can automatically learn hierarchical visual representations and have been widely applied to agricultural tasks involving crop detection, disease identification, weed recognition, fruit counting, crop phenotyping, and robotic harvesting [10,15,16]. Among these approaches, one-stage object detectors from the You Only Look Once (YOLO) family have received considerable attention because they provide a useful balance between localization accuracy and computational speed [16,17,18,19]. This characteristic is particularly advantageous for agricultural monitoring systems that must process sequential images or continuous video rather than isolated images.

The development of recent YOLO architectures has further improved multi-scale feature extraction and real-time object detection [18,19]. Different versions of the YOLO model have been applied to several agricultural detection tasks because of its relatively efficient architecture and suitability for transfer learning [17,18]. Recent studies have demonstrated the potential for detecting crops and plant organs under complex field conditions. For example, Ning et al. [20] developed an improved YOLOv8 approach for corn leaf detection and counting, while Zhao et al. [21] applied an improved YOLOv8 model to soybean flower and pod detection. Li et al. [22] used an improved YOLOv8 model for rice seedling density estimation, and Solimani et al. [23] investigated modifications to YOLOv8 for tomato plant phenotyping. Such studies have demonstrated the suitability of YOLOv8 and related architectures for agricultural object detection, while also indicating that crop-specific modifications are often required to address variations in object scale, occlusion, and field background complexity. Task-specific detector refinement has also been investigated in other sensing domains. Lv et al. [24] incorporated dynamic upsampling and feature-enhancement mechanisms into YOLOv11 for underground pipeline recognition in radar images. This provides methodological context for detector adaptation, although its findings do not establish performance for RGB crop imagery.

For cabbage, Jiang et al. [25] proposed an improved YOLOv8n-based field detection and positioning system for variable illumination and natural environments. Zheng et al. [13] addressed cabbage-head keypoint detection and diameter estimation under different occlusion levels, and Fu et al. [26] investigated the effect of light occlusion on online cabbage recognition. At the seedling stage, Chen et al. [10] developed a TSP-YOLO approach for UAV-based cabbage seedling emergence monitoring by combining an improved YOLOv8n detector with tracking, whereas Zhao et al. [14] investigated lightweight UAV-based detection and counting of early-stage cabbage seedlings. Related studies on flowering Chinese cabbage have also applied deep learning to plant and organ counting. Yuan et al. [27] improved YOLOv5 feature fusion for bud detection and counting, Wu et al. [28] developed a lightweight maturity-detection and counting framework, and Shui et al. [29] combined an improved YOLOv8 model with ByteTrack for multi-target detection and positioning. These studies collectively highlight persistent challenges associated with small targets, dense planting, similar plant appearance, occlusion, and variable field conditions.

Previous cabbage studies have addressed different developmental stages and operational objectives. Seedling-oriented studies have investigated emergence monitoring and stand establishment [10,14], whereas other studies have examined cabbage recognition, harvesting-related detection, positioning, and occlusion handling [13,25,26]. These applications provide a foundation for automated cabbage monitoring, but frame-wise detection performance alone does not establish reliable cumulative counting from moving-camera videos. The same stationary plant may appear repeatedly across consecutive frames, while temporary occlusion or missed detection may interrupt its trajectory. Evaluating detection together with temporal association and counting is therefore necessary to assess video-based population monitoring. This study addresses these linked requirements for seedlings and grown plants within the sampled greenhouse and open-field conditions.

Tracking-by-detection enables repeated frame-wise detections to be associated with persistent plant identities. DeepSORT uses appearance descriptors for identity association [30,31], whereas ByteTrack improves trajectory continuity by retaining lower-confidence detections during association [32]. These methods have been applied to agricultural counting, including cotton seedlings [33,34] and missing rice seedlings [35]. However, crop-row tracking is particularly challenging because plants are stationary while the camera moves, and neighboring plants often have similar appearances. Occlusion, motion blur, and temporary missed detections can therefore cause identity fragmentation and repeated counting. BoT-SORT is well suited to moving-camera applications because it integrates motion prediction, appearance information, and camera-motion compensation [36]. Nevertheless, fragmented tracks can still generate duplicate plant counts.

Previous agricultural applications have demonstrated the value of integrating object detection and tracking for crop monitoring [10,28,36,37]. However, frame-wise detection and cumulative video counting address different requirements: plants must first be detected and then associated across frames to limit missed or repeated counting. This study therefore evaluates an integrated framework for cabbage seedlings and grown plants under the sampled greenhouse and open-field conditions. Its contribution lies in the task-specific integration and evaluation of established components.

To address variation in plant size, overlapping foliage, and background appearance, CBAM was selectively incorporated into the YOLOv8n neck to refine channel and spatial features [38,39]. These conditions motivated the design; the contribution of attention was assessed through channel-only, spatial-only, and complete CBAM comparisons. To address interrupted trajectories and repeated counting candidates during camera movement, baseline BoT-SORT [35] was extended with minimum trajectory persistence, short-gap relinking, class-consistent association, and duplicate-event filtering. ByteTrack [31], DeepSORT [29], and baseline BoT-SORT [35] were included as comparison methods. The proposed tracking modifications were evaluated jointly, and their individual contributions were not isolated.

Computational efficiency is also relevant to practical crop monitoring [14,20,22,27]. Accordingly, this study evaluated detection accuracy, cumulative counting, trajectory performance, and computational performance separately. The objective was to develop and assess an RGB image- and video-based framework for detecting and counting cabbage seedlings and grown plants within the collected dataset, while distinguishing the effects of detector modification from those of the complete tracking procedure.

## 2. Materials and Methods

### 2.1. Data Collection Sites

**Figure 1:**
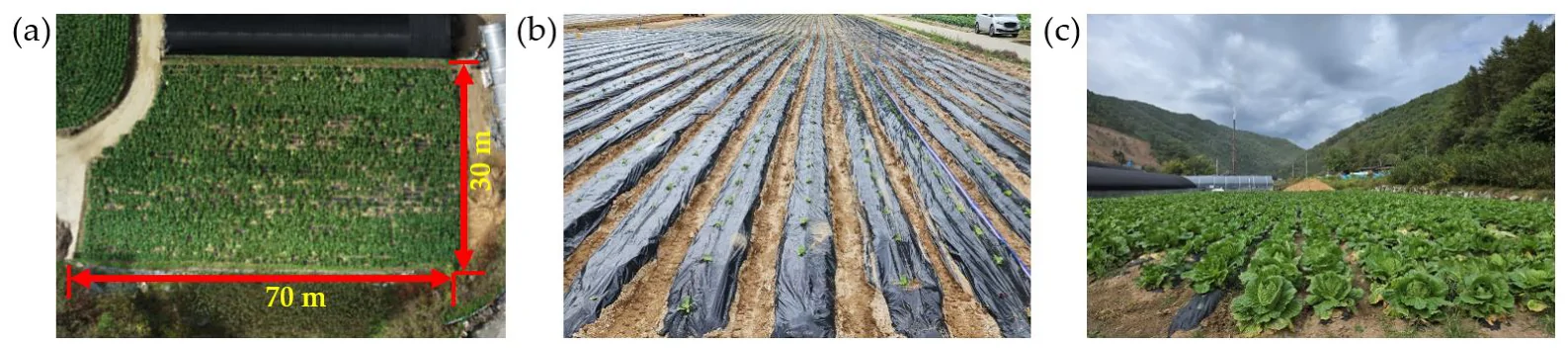
Data collection site in an open-field cabbage cultivation area: (**a**) field size from the top view, (**b**) cabbage field during the transplanting stage, and (**c**) cabbage field during the growth and harvesting stage.

The study was conducted in two distinct cultivation environments: an open field, and a greenhouse. The use of two cultivation systems was intended to provide a diverse dataset reflecting practical cabbage production conditions, including differences in background complexity, illumination, row structure, and canopy development. The open-field experiments were carried out in Hongcheon, Gangwon-do, Republic of Korea, across a cabbage plot covering an area of approximately 70 m × 30 m, as shown in Figure 1a. Figure 1b presents the field condition during the transplanting stage, when cabbage seedlings were newly established in the ridged and plastic-mulched rows. Figure 1c shows the same field during the later growth and harvesting stage, where the crop canopy became more developed and spatial plant distribution was clearly visible. This site represented typical ridge-type open-field cultivation conditions common in the region. Field measurements were conducted at two crop stages. Transplanting-related data were collected on 23 July 2024, immediately after the transplanting operation, whereas measurements of fully developed cabbage plants were obtained on 29 October 2024, immediately before harvesting.

**Figure 2:**
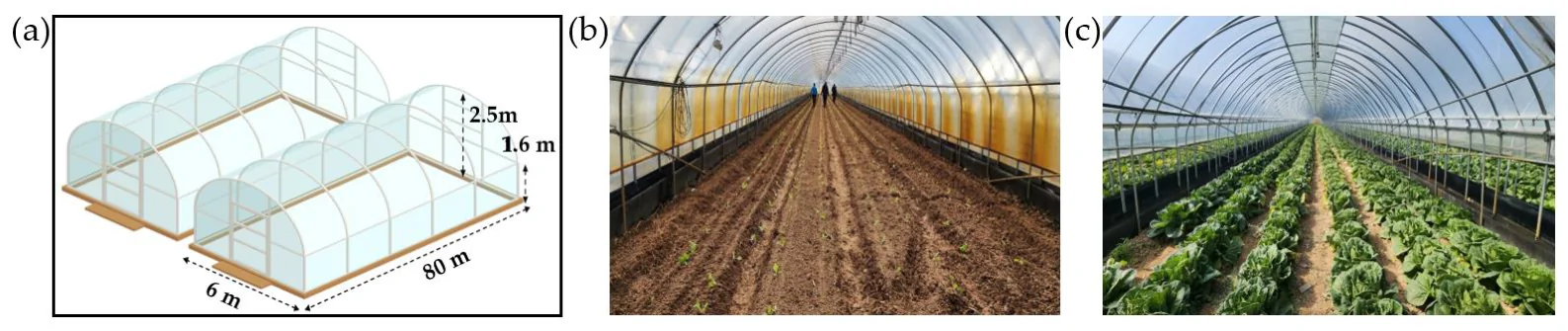
The data collection sites using a greenhouse cabbage cultivation area: (**a**) schematic diagram of the greenhouse, (**b**) greenhouse during the transplanting time, and (**c**) greenhouse during the growth and harvesting time.

The greenhouse experiments were conducted in Yeasan, Republic of Korea, within a greenhouse cultivation structure measuring approximately 80 m × 6 m × 2.5 m, as shown in Figure 2a. Field data were collected at two representative crop stages to reflect different plant and background conditions. Figure 2b shows the greenhouse during the transplanting stage, when cabbage seedlings were sparsely distributed and the soil background was dominant. Figure 2c presents the greenhouse during the growth and harvesting stage, when canopy coverage increased substantially and plant rows became visually denser. These stage-specific images were included to capture variations in plant size, spacing, occlusion, and illumination conditions within the greenhouse environment. Measurements were performed at two distinct growth stages. Greenhouse data were acquired on 4 February 2025 immediately after transplanting and on 2 May 2025 shortly before harvesting.

Data collection in both the open field and greenhouse was initiated immediately after the transplanting process was completed and before harvesting, allowing for close observation of cabbage seedlings and plants under original field conditions. Together, these two experimental sites provided complementary datasets representing both open-field and greenhouse production systems, ensuring that the proposed detection and counting methodology could be validated under diverse cultivation environments. Greenhouse and open-field recordings were acquired at different sites and dates; therefore, cultivation environment was confounded with site, year, season, and potentially crop-management conditions. Comparisons between these environments were descriptive and were not designed to isolate an environmental effect.

### 2.2. Data Collection and Processing Procedures

**Figure 3:**
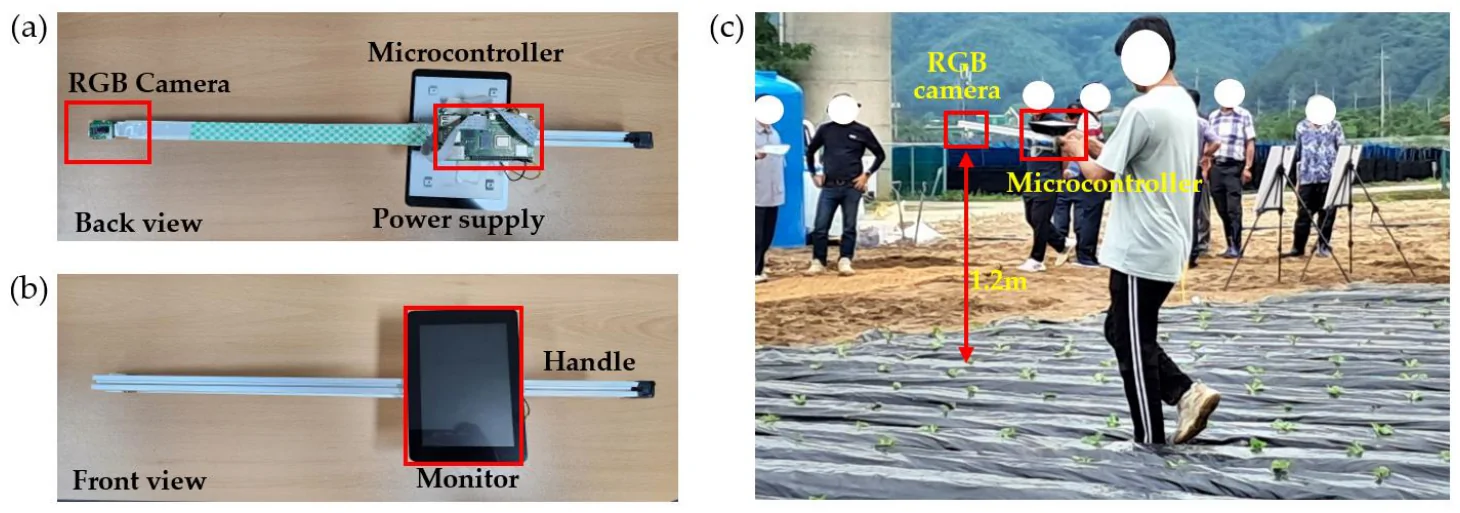
Portable RGB imaging system developed for cabbage data collection: (**a**) overall configuration showing the RGB camera, microcontroller, and power supply, (**b**) front-side view with the integrated monitor and handle, and (**c**) field operation during data acquisition in the cabbage cultivation area.

The RGB imaging module used for cabbage detection and counting was developed as a portable device that combined a low-cost, portable, and high-quality RGB camera (model: Raspberry Pi Camera V2, Raspberry Pi Foundation, Cambridge, UK) with a microcontroller (model: Raspberry Pi 4 Model B, Raspberry Pi Foundation, Cambridge, UK) powered by a rechargeable lithium-ion battery pack. The specifications of the microcontroller and camera are listed in Table 1. The camera was mounted at the front end of a lightweight aluminum frame, while the microcontroller and power supply were fixed at the base to maintain balance and stability during operation, as shown in Figure 3a,b. A monitor was integrated into the frame to provide real-time visual feedback, allowing the operator to check video and image quality, monitor module performance, and ensure consistent coverage of the target plants during data acquisition. For field deployment, the device was manually operated and positioned at a height of approximately 1.2 m above the canopy to maximize the field of view (FOV) and capture multiple rows of cabbage seedlings within a single frame, as shown in Figure 3c. This elevated configuration minimized occlusion effects from overlapping leaves and provided uniform video coverage across the cultivation area. The module was designed to be portable, with a handle allowing smooth movement across both greenhouse and open-field plots. The microcontroller handled video acquisition, temporary storage on a 32 GB microSD card, and power management, enabling continuous operation for up to 8 h per charge. This low-cost, flexible imaging setup provided an efficient platform for generating high-quality RGB datasets required for training and validation of deep learning models aimed at automated detection and counting of cabbage seedlings and mature plants under diverse cultivation conditions.

Data were collected using the portable RGB sensor platform in both open-field and greenhouse cabbage cultivation areas. During acquisition, the sensor was manually operated at a height of approximately 1.2 m above the seedling or plant canopy to capture multiple rows within the field of view while reducing leaf occlusion. The platform was moved at a steady speed of about 1.0 ms^−1^, corresponding to normal human walking speed, to maintain stable image continuity and consistent acquisition conditions. Furthermore, the spatial coverage of the sensor was considered using Equation (1), which was used to estimate the ground sampling distance covered by the camera based on the sensing geometry.

(1) $$W=2h\times \tan\left(\frac{\theta_{H}}{2}\right),\mathrm{GS}D_{H}=\frac{W}{N_{H}}$$

where W is horizontal coverage at the reference plane, h is the perpendicular camera to plane distance, θ_H_ is horizontal field of view, and N_H_ is the horizontal image resolution. These acquisition conditions were selected to ensure uniform, repeatable, and high-quality RGB data for cabbage detection and counting under different crop growth stages and cultivation environments.

Video data were recorded continuously during operation from the open field and greenhouse and were subsequently converted into image frames at a rate of 30 fps for further processing. From the extracted image set, frames were randomly selected for annotation in order to construct the dataset used for model training and validation. Random image selection was performed to avoid bias toward specific field locations, crop densities, or visual conditions, thereby ensuring that the annotated dataset included diverse examples from different cultivation environments and growth stages.

Images were manually annotated using MakeSense.ai with two classes: cabbage seedling, and grown cabbage plant. Class labels were assigned according to the growth stage recorded during acquisition and checked against plant appearance. Each distinguishable plant was enclosed by an axis-aligned bounding box covering its visible extent. Partially occluded plants and plants intersecting image boundaries were included when their identity and class could be determined reliably; bounding boxes were restricted to the image boundaries. Fully occluded plants were not annotated. Ambiguous instances were reviewed using the corresponding source-video frames. Images with unresolved ambiguities were excluded before dataset partitioning.

One annotator prepared the initial labels, and a second annotator reviewed all labeled images for missing plants, incorrect classes, duplicate boxes, and inaccurate box placement. Disagreements were resolved through joint inspection, and corrected annotations were checked again. Automated checks identified invalid class indices, non-positive box dimensions, coordinates outside image boundaries, and missing or unreadable label files. The same annotation rules were applied across both cultivation environments and all dataset partitions. Image and annotated-instance distributions are reported in Appendix A, Table A1.

**Figure 4:**
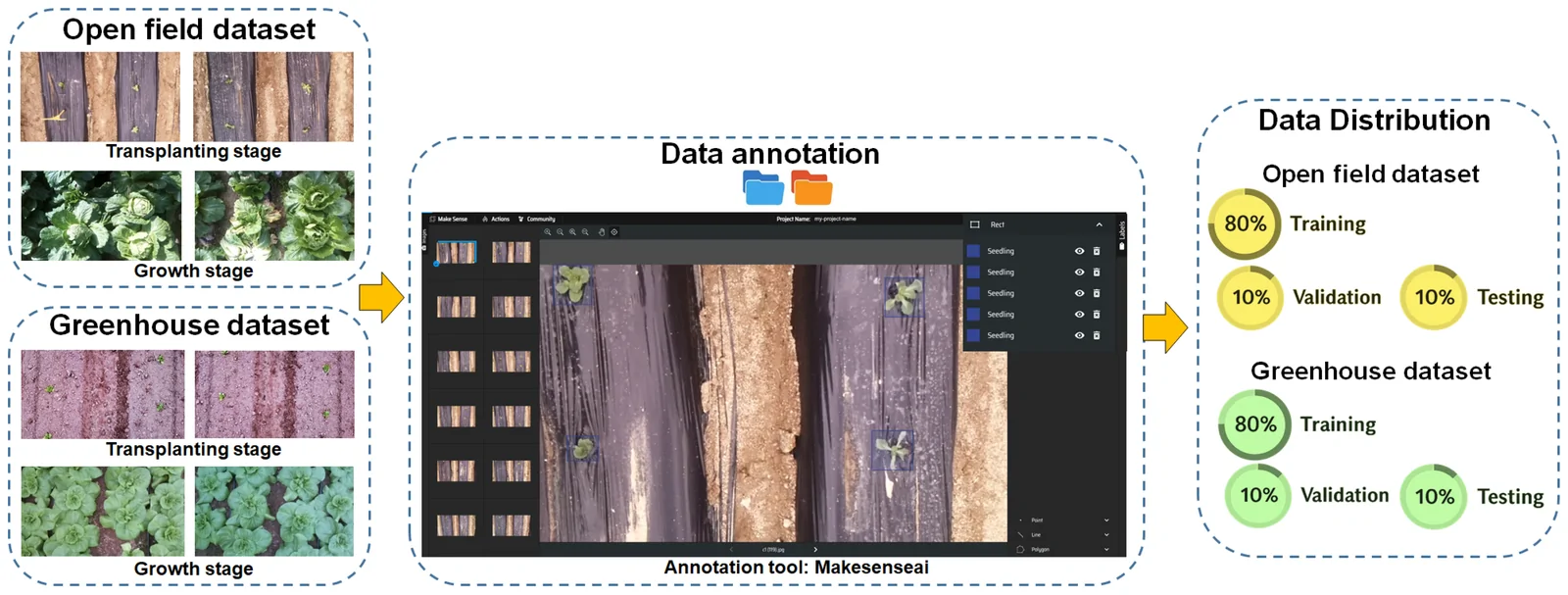
Dataset preparation workflow for cabbage seedling and plant detection and counting, showing image collection from open-field and greenhouse datasets, manual annotation using MakeSense.ai (https://www.makesense.ai/; accessed on 12 June 2026), and distribution into training, validation, and test sets.

The annotated dataset was divided into independent training, validation, and test sets to avoid data leakage during model training and evaluation, as shown in Figure 4. The final dataset comprised 1940 images and 7498 annotated plant instances, including 970 images from each cultivation environment. Dataset partitioning was performed at the video/acquisition-session level, with images from each acquisition group assigned exclusively to one subset. Overlapping views were carefully screened to ensure that the same physical plants did not occur across training, validation, and test subsets. For each environment, 776 images were allocated to training, 97 to validation, and 97 to testing, yielding combined totals of 1552, 194, and 194 images, respectively. Data augmentation was restricted to the training subset.

For each detector configuration, a single model was trained using the combined greenhouse and open-field training images, including both seedlings and developed cabbage plants. Detection performance was evaluated on the combined held-out test set and separately for each environment–growth-stage subset. The same trained checkpoint was used for all subset evaluations, without environment-specific retraining. The detector models were developed using the image dataset, whereas the nine independent videos were reserved exclusively for final tracking and counting evaluation. No images extracted from these videos were used for detector training, model selection, or tracker-parameter optimization.

The nine videos used for tracking and counting evaluation were independent of the detector-development dataset. Their acquisition sites, dates, cultivation environments, growth stages, frame counts, durations, and manual reference counts are summarized in Appendix A, Table A2. Predictions and video-level counting errors for each evaluated configuration are provided in Appendix A, Table A3. These records allow the aggregate counting metrics to be interpreted alongside variation among individual recordings.

**Figure 5:**
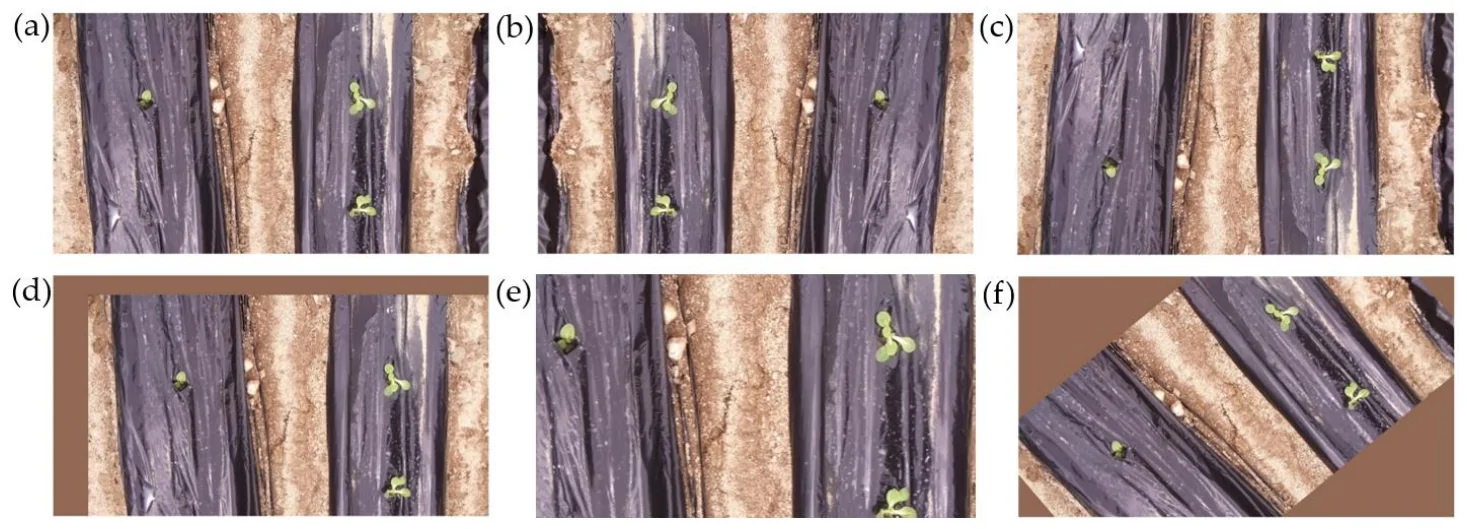
Image augmentation: (**a**) original image, (**b**) horizontal flip, (**c**) vertical flip, (**d**) shift, (**e**) zoom, and (**f**) rotation.

To improve model robustness and mitigate overfitting, data augmentation was incorporated into the YOLOv8 training pipeline. The applied augmentation operations included horizontal and vertical flipping, translation, shift, zoom and rotation, thereby increasing the variability of the training samples and enhancing the model ability to generalize across different cultivation conditions, as shown in Figure 5. These transformations were effective in addressing variations in plant orientation, spatial arrangement, scale, background complexity, and partial occlusion. In addition, transfer learning was performed by initializing the YOLOv8 model with weights pre-trained on the COCO dataset. This strategy provided strong initial feature representations, accelerated the optimization process, and improved detection performance under limited training data conditions. Consequently, the final data processing procedure supported robust training and reliable evaluation of the proposed YOLOv8-based cabbage seedling and plant detection framework.

### 2.3. Cabbage Seedling and Plant Detection Model

#### 2.3.1. YOLO V8 Model

**Figure 6:**
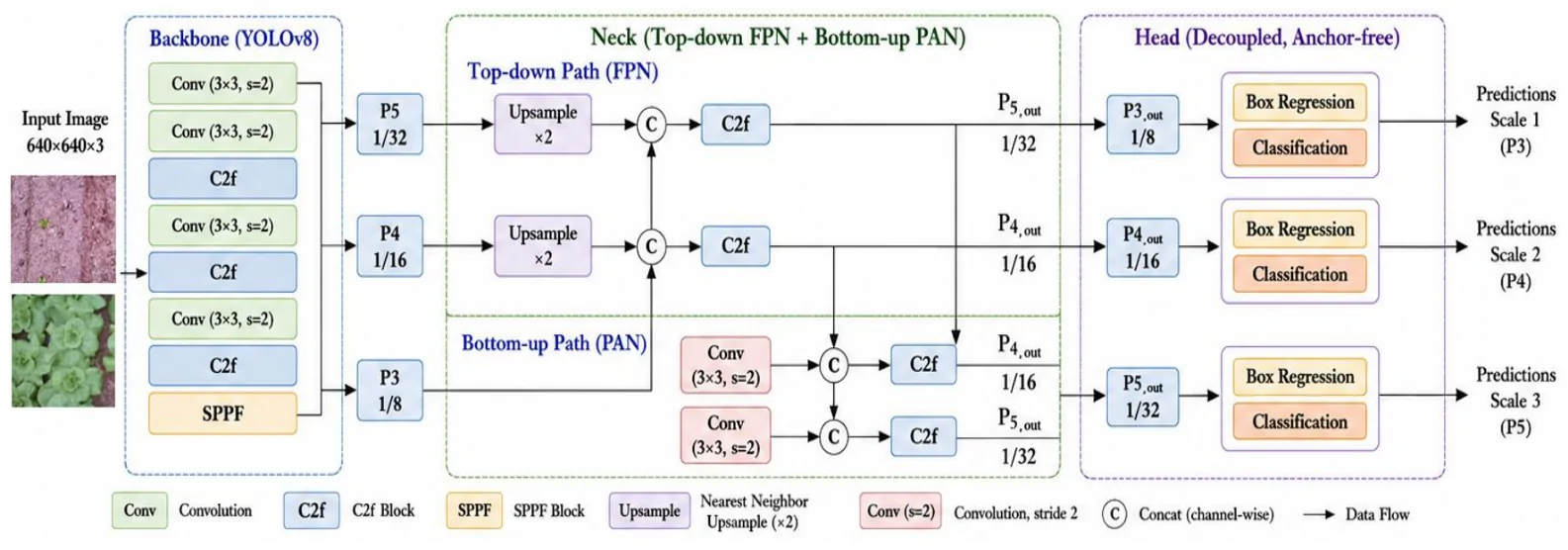
Baseline YOLOv8 architecture used for cabbage seedling and cabbage plant detection, showing the C2f/SPPF backbone, FPN–PAN multi-scale feature-fusion neck, and decoupled anchor-free detection head. The three output scales, P3, P4, and P5, are used jointly for localization and classification of cabbage plants at different apparent sizes.

YOLOv8n was adopted as the baseline object-detection model because it provides a practical balance between detection accuracy and computational efficiency, which is important for processing continuous RGB images and video acquired under field conditions. In this study, the detector was trained to distinguish two target classes, namely cabbage seedlings and grown cabbage plants. The YOLOv8 architecture can be broadly divided into three functional components: the backbone, neck, and detection head [40] as shown in Figure 6. The backbone extracts hierarchical visual features from the input image, the neck combines information from different feature scales, and the detection head performs object localization and classification. Compared with earlier YOLO architectures, YOLOv8 employs C2f modules and an anchor-free detection strategy, enabling efficient feature propagation while avoiding the need to predetermine anchor-box dimensions [40,41].

The backbone progressively transforms the input RGB image into feature maps with increasing semantic information and decreasing spatial resolution. Convolutional layers with a stride of two are used for spatial downsampling, while C2f (Cross-Stage Partial with two convolutions) modules constitute the principal feature-extraction blocks. The C2f structure divides and subsequently aggregates feature information through multiple bottleneck paths, thereby promoting feature reuse and gradient propagation without introducing excessive computational complexity. This characteristic is particularly relevant to the present application because the visual appearance of cabbage changes considerably between the transplanting and mature growth stages. Newly transplanted seedlings generally occupy relatively few pixels and contain limited leaf information, whereas grown cabbage plants exhibit larger and more complex canopy structures.

At the deepest part of the backbone, a Spatial Pyramid Pooling–Fast (SPPF) module is used to increase the effective receptive field and aggregate contextual information at multiple spatial scales. Instead of relying solely on local leaf characteristics, this operation enables the network to incorporate broader contextual information surrounding a candidate plant. Such information can be useful under field conditions where cabbage plants must be differentiated from soil, plastic mulch, shadows, weeds, and neighboring vegetation. The backbone consequently provides feature maps at different spatial resolutions, conventionally represented by P3, P4, and P5, corresponding approximately to downsampling factors of 8, 16, and 32 relative to the input image.

The neck performs multi-scale feature aggregation by combining deep semantic information with higher-resolution spatial information. A top-down feature-pyramid pathway first upsamples deeper features and concatenates them with corresponding features from earlier backbone stages. This process helps recover spatial details that may otherwise be weakened during successive downsampling operations. A subsequent bottom-up path aggregation pathway further combines the fused features and strengthens information exchange among different resolution levels. The resulting P3, P4, and P5 feature maps are therefore derived from both spatially detailed shallow features and semantically rich deep features. Multi-scale fusion is particularly important in this study because a single detector is required to recognize small cabbage seedlings as well as substantially larger grown plants.

The fused feature maps are subsequently passed to the anchor-free decoupled detection head. Unlike anchor-based YOLO detectors, YOLOv8 directly estimates object locations without matching predictions to a predefined set of anchor boxes. Moreover, localization and classification are processed through separate branches of the detection head. The regression branch estimates the spatial extent of each cabbage plant, whereas the classification branch determines whether the detected object belongs to the seedling or grown cabbage class. Predictions are generated from the three feature scales, allowing objects with substantially different apparent sizes to be represented within the same detection framework. It should be noted that P3, P4, and P5 are not assigned exclusively to particular crop classes; rather, detections from all three scales collectively contribute to both classes.

The models were trained using stochastic gradient descent (SGD). The total detection loss was calculated using stochastic gradient descent (SGD) to minimize the detection loss. For YOLOv8, the overall detection loss comprises bounding-box regression, classification, and distribution focal loss (DFL) components and can be expressed in generalized form as Equation (2).

*L_yolo_* = *λ_box_L_box_* + *λ_cls_L_cls_* + *λ_DFL_L_DFL_* (2)

where L_box_ represents the IoU-based bounding-box regression loss, L_cls_ represents the classification loss, and L_DFL_ denotes the distribution focal loss (DFL). The coefficients λ_box_, λ_cls_, and λ_DFL_ control the relative contribution of each component during optimization.

The overlap between a predicted bounding box B_p_ and its corresponding ground-truth box B_g_ can be described using intersection over union (IoU) as shown in Equation (3).

(3) $$\mathrm{IoU}=\frac{\left|B_{p}\cap B_{g}\right|}{\left|B_{p}\cup B_{g}\right|}$$

YOLOv8 uses an IoU-based localization objective together with DFL to improve bounding-box estimation. DFL treats the distances defining the four sides of a bounding box as discrete probability distributions rather than directly regressing only continuous scalar offsets. This distribution-based representation provides finer localization information around object boundaries and was originally introduced as part of Generalized Focal Loss [42]. This feature can be beneficial for cabbage imagery because the visible boundaries of seedlings and mature canopies can vary substantially with growth stage, leaf orientation, occlusion, and viewing geometry.

The classification component evaluates the correspondence between the predicted class probability and the ground-truth class label. For the two-class problem considered in this study, the model learns class-specific responses for seedlings and grown cabbage plants. During inference, the outputs from the three detection scales are converted into bounding-box coordinates and associated class-confidence scores. Predictions below the selected confidence threshold are removed, and overlapping predictions are filtered according to the inference configuration before the remaining detections are transferred to the multi-object tracking and counting stage.

The baseline YOLOv8 architecture therefore provides a computationally efficient framework for detecting cabbage plants across markedly different growth stages. Nevertheless, field-acquired images contain considerable background variation, and the standard feature-fusion process does not explicitly determine which channels or spatial regions contain the most informative crop features. Small seedlings may be represented by weak features relative to the surrounding soil or plastic mulch, while mature plants may experience substantial inter-plant leaf overlap. To address these limitations, the baseline architecture was further modified by incorporating a Convolutional Block Attention Module (CBAM) into selected multi-scale feature-fusion stages of the YOLOv8 neck, as described in the following section.

#### 2.3.2. Improved Yolov8 Model

Although the baseline YOLOv8 model provides efficient multi-scale object detection, cabbage detection presents substantial variations in target size and visual characteristics across growth stages. Newly transplanted cabbage seedlings occupy a relatively small image area and can exhibit weak visual contrast against soil, plastic mulch, crop residues, and shadows. In contrast, grown cabbage plants contain larger and more complex leaf structures, and adjacent plants may partially overlap as canopy development progresses. Consequently, features relevant to cabbage detection may compete with background responses during multi-scale feature fusion. To improve the discrimination of crop-related features while maintaining the computationally efficient structure of YOLOv8, a modified YOLOv8-CBAM architecture was developed in this study.

The proposed architecture preserves the backbone, Spatial Pyramid Pooling–Fast (SPPF) module, bottom-up path aggregation, and anchor-free decoupled detection head of the baseline YOLOv8 model. The principal modification is made in the top-down feature-fusion pathway of the neck, where two Convolutional Block Attention Modules (CBAMs) are introduced after selected C2f blocks. CBAM is a lightweight attention mechanism that sequentially recalibrates a feature tensor along its channel and spatial dimensions [39]. Rather than treating every extracted feature with equal importance, the module assigns larger weights to informative feature responses and smaller weights to less relevant responses.

The modification was restricted to the feature-fusion neck instead of inserting attention modules throughout the entire backbone. This design was selected to retain the original feature-extraction capability of YOLOv8 while refining the multi-scale features after shallow spatial information and deeper semantic information have been combined. In particular, the first CBAM operates on the feature obtained after fusion of the upsampled P5 feature with the corresponding P4 backbone feature. The second CBAM is applied after the subsequent fusion with the higher-resolution P3 feature. Thus, the modified top-down pathway can be represented using Equation (4).

(4) $$P_{5}\overset{\mathrm{UP}}{\to }\;\mathrm{Concat}\;(P_{4})\overset{C2f}{\to }\;\mathrm{CBAM}\;\overset{\mathrm{UP}}{\to }\;\mathrm{Concat}\;\left(P_{3}\right)\;\overset{C2f}{\to }\;\mathrm{CBAM}$$

The resulting high-resolution attention-refined feature is subsequently passed through the original bottom-up path aggregation process to generate the final multi-scale features $P_{3}^{\mathrm{out}}$, $P_{4}^{\mathrm{out}}$, $P_{5}^{\mathrm{out}}$. These feature maps are supplied to the unchanged decoupled detection head for bounding-box regression and classification into the two target categories: seedling, and grown cabbage. Therefore, the difference between the baseline and proposed models is intentionally limited to attention-assisted feature refinement in the neck, allowing the contribution of CBAM to be evaluated directly.

**Figure 7:**
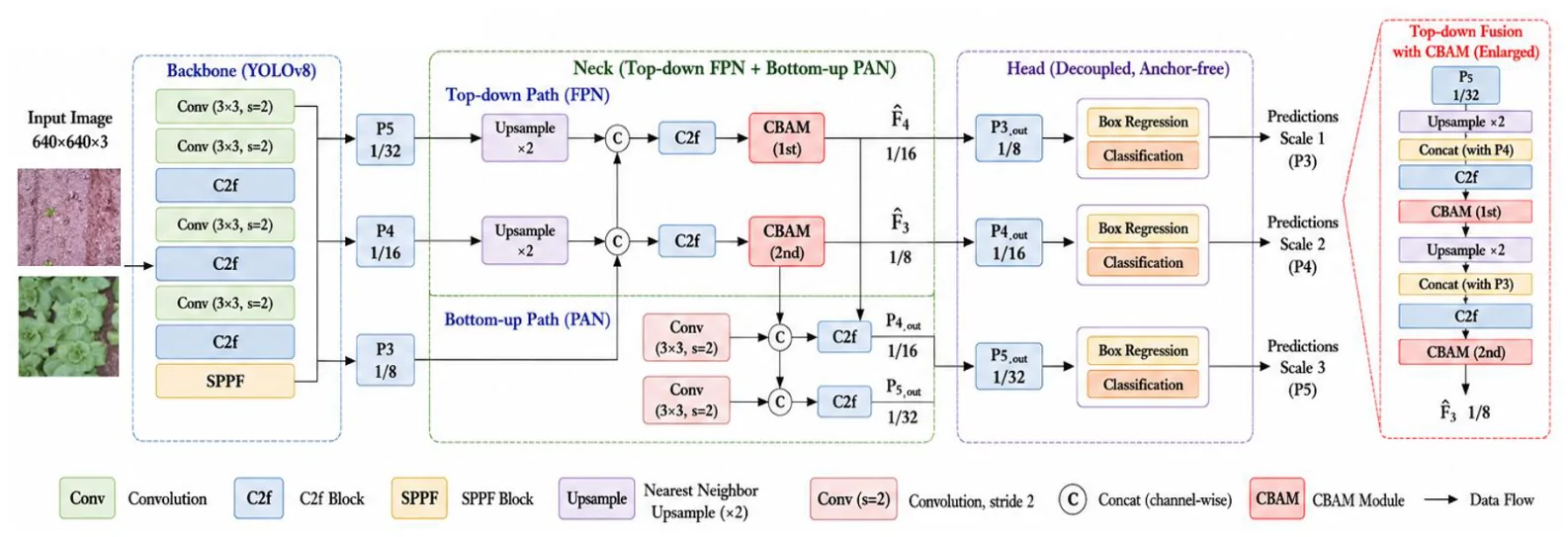
Proposed YOLOv8-CBAM architecture with two CBAM modules incorporated into the top-down feature-fusion pathway of the neck. The attention-refined features are subsequently aggregated through the original bottom-up PAN pathway and supplied to the P3, P4, and P5 detection scales for cabbage seedling and grown-cabbage detection.

The rationale for this modification is related to the characteristics of the cabbage dataset. Higher-resolution fused features retain detailed spatial information that is important for detecting small seedlings, whereas the integration of deeper features provides semantic information required to distinguish developed cabbage canopies from complex backgrounds. Applying attention after these fusion operations allows the network to recalibrate the combined representations before they are propagated to the subsequent detection scales. The proposed modification as shown in Figure 7, is therefore intended to strengthen crop-relevant features while reducing the influence of background patterns and less informative feature responses. Whether this architectural change improves detection accuracy is evaluated experimentally by comparing the baseline YOLOv8 and proposed YOLOv8-CBAM models under identical training and testing conditions.

#### 2.3.3. Convolutional Block Attention Module

**Figure 8:**
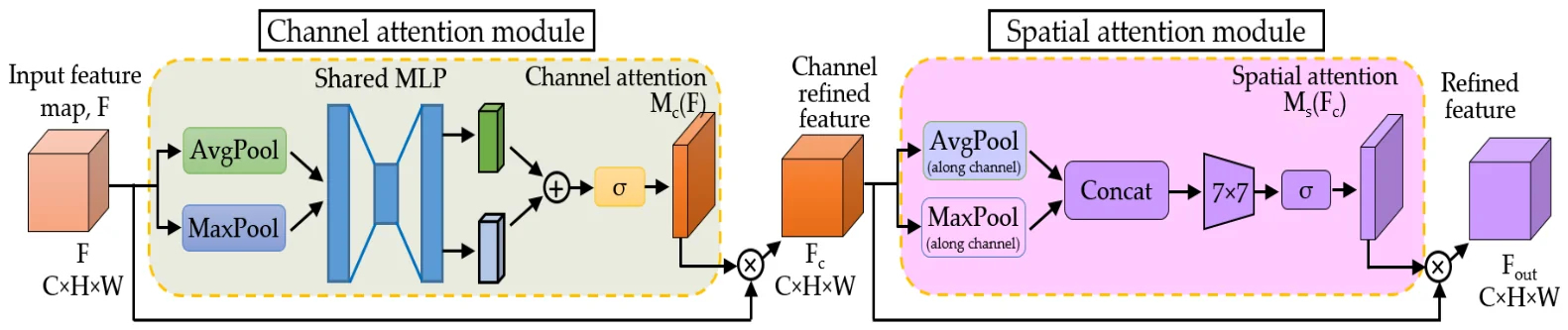
Structure of the CBAM used in the proposed YOLOv8-CBAM model. An input feature map is first refined by channel attention and subsequently by spatial attention. The two attention maps are sequentially multiplied with the intermediate features to produce the final refined representation.

The Convolutional Block Attention Module was originally proposed as a lightweight feature-refinement mechanism that can be integrated into convolutional neural networks with relatively limited additional computational complexity [39] as shown in Figure 8. CBAM applies two consecutive attention operations: channel attention followed by spatial attention. The channel-attention stage determines which feature channels should receive greater emphasis, whereas the spatial-attention stage determines where informative responses are located within the feature map. The sequential combination of these operations enables feature recalibration in both the channel and spatial dimensions.

For an input feature map F, CBAM sequentially applies channel attention and spatial attention. The channel-attention module determines the relative importance of individual feature channels. Global average pooling and maximum pooling are applied to the input feature map, and the resulting descriptors are processed using a shared multilayer perceptron (MLP). The channel-attention map is calculated using Equation (5).

M_c_(F) = σ[MLP(AvgPool(F)) + MLP(MaxPool(F))] (5)

The channel-refined feature map is then obtained as Equation (6).

F_c_ = M_c_(F)⊗F (6)

where M_c_(F) represents the channel-attention map, σ is the sigmoid activation function, and ⊗ denotes element-wise multiplication. This operation adaptively assigns different weights to individual feature channels according to their relative importance.

The spatial-attention module subsequently determines the important spatial regions within the channel-refined feature map F_c_. Average pooling and maximum pooling are performed along the channel dimension, and the resulting feature maps are concatenated. A 7 × 7 convolution followed by sigmoid activation is then used to generate the spatial-attention map using Equation (7).

*M_s_*(F_c_) = σ{*f*^7×7^[*AvgPool_c_*(*F_c_*);*MaxPool_c_*(*F_c_*)]} (7)

where M_s_(F_c_) denotes the spatial-attention map and f^7×7^ represents a convolution operation with a 7 × 7 kernel. Thus, channel attention determines which feature channels should be emphasized, whereas spatial attention determines where the informative features are located. In the proposed YOLOv8-CBAM architecture, this sequential attention mechanism is applied after the two selected C2f blocks in the top-down feature-fusion pathway of the neck.

#### 2.3.4. Model Training and Implementation

The baseline YOLOv8n and proposed YOLOv8-CBAM models were trained using identical dataset partitions and training conditions to ensure a fair comparison. Transfer learning was performed using COCO-pretrained YOLOv8 weights, while the newly introduced CBAM layers were initialized and optimized during training. Data augmentation, including horizontal and vertical flipping, translation/shift, zoom, and limited rotation, was applied only to the training dataset to improve model robustness against variations in plant orientation, scale, illumination, and field background.

Model optimization was performed using stochastic gradient descent (SGD). The overall YOLOv8 detection loss consisted of bounding-box regression, classification, and Distribution Focal Loss (DFL) components, as defined in Equation (2). Training convergence was monitored using the training and validation classification losses together with precision, recall, mAP@0.5, and mAP@0.5:0.95. Precision–recall and F1–confidence curves were also generated to examine model performance at different confidence thresholds. The model checkpoint achieving the best validation performance was retained for final evaluation and subsequently used for image- and video-based detection, tracking, and counting.

The baseline and proposed models were implemented using Python (ver. 3.12), PyTorch (ver. 2.5.1), and the Ultralytics (ver. 8.3) YOLO framework. GPU acceleration was used when available. Identical training parameters were maintained for the baseline and proposed models so that differences in detection performance could be primarily attributed to the architectural modification. Each detector configuration was trained independently using five random seeds (42, 43, 44, 45, and 46). Acquisition-group partitions, pretrained initialization source, augmentation settings, maximum number of training epochs, early-stopping settings, and checkpoint-selection criteria were kept consistent across configurations. For each run, the checkpoint was selected according to validation performance and evaluated on the same fixed held-out test subset. Precision, recall, mAP@0.50, and mAP@0.50:0.95 were reported as the mean ± sample standard deviation across the five runs. Test performance was not used to select seeds or checkpoints. The principal model-training parameters and computing environment are summarized in Table 2.

### 2.4. Cabbage Seedling and Plant Tracking

Detection of individual plants in isolated video frames does not directly provide a reliable cumulative plant count because the same cabbage may remain visible across several consecutive frames. Therefore, a tracking-by-detection framework was employed to associate detections temporally and assign a persistent identity to each plant. The trained YOLOv8-CBAM detector first generated bounding boxes, class labels, and confidence scores for cabbage seedlings and grown cabbage plants in each video frame. These detections were subsequently supplied to a multi-object tracker, which maintained plant identities across consecutive frames and generated individual trajectories.

BoT-SORT was selected as the baseline tracker because it integrates motion prediction, detection association, appearance information, and camera-motion compensation within a unified tracking framework [36]. These characteristics are relevant to the present application because the RGB imaging system was manually moved over the cabbage rows, resulting in global image displacement in addition to the apparent motion of individual plants. Short-term occlusion, illumination variation, repetitive plant appearance, and overlapping leaves further increase the possibility of fragmented trajectories and duplicate counting.

**Figure 9:**
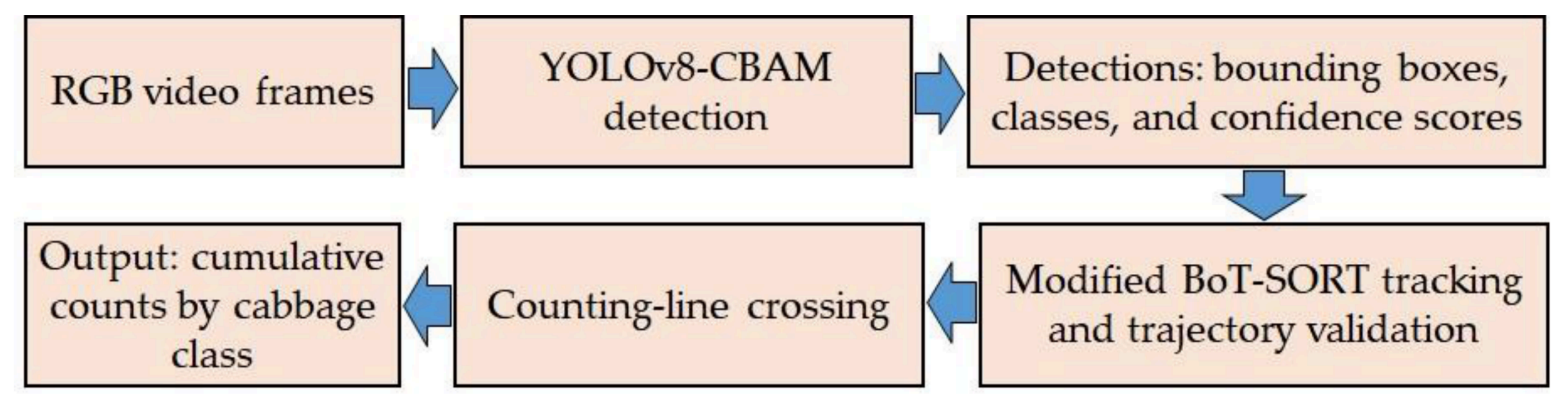
Overall cabbage detection and counting framework. YOLOv8-CBAM detections are processed by the modified tracking procedure, and eligible trajectories are converted into cumulative counts of cabbage seedlings and grown cabbage plants.

The overall tracking and counting procedure as shown in Figure 9, consisted of five principal operations: (1) frame-wise cabbage detection using YOLOv8-CBAM, (2) motion prediction and camera-motion compensation, (3) association of detections with existing tracks, (4) track management and identity preservation, and (5) trajectory-based counting across a predefined counting line.

#### 2.4.1. Baseline BoT-SORT Multi-Object Tracking

**Figure 10:**
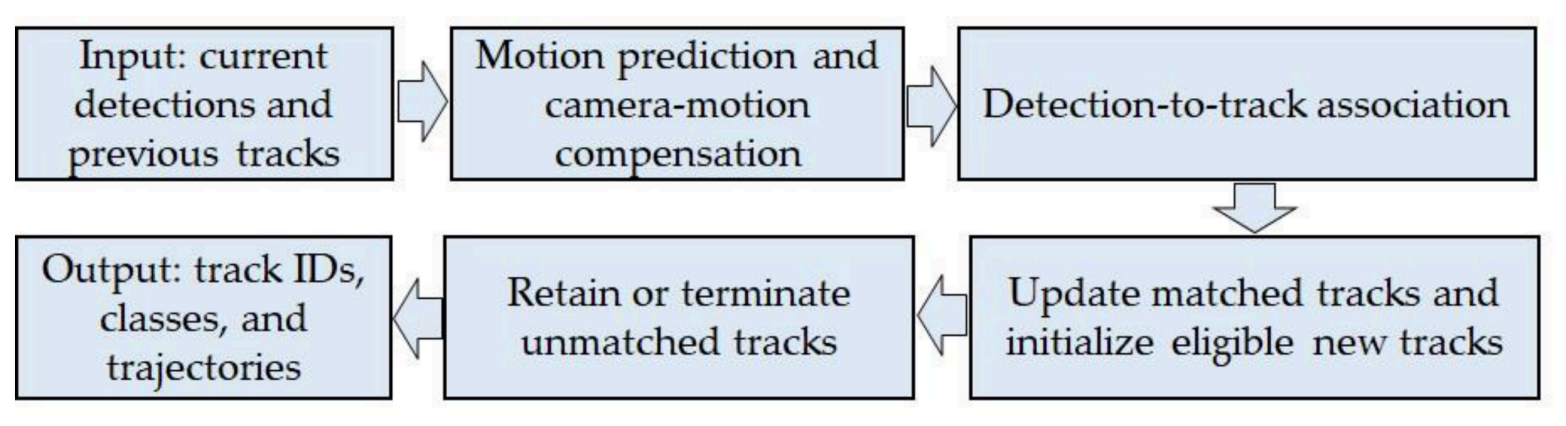
Baseline BoT-SORT tracking workflow, including motion prediction, camera-motion compensation, detection association, and track management. The resulting track identities and trajectories are supplied to the counting procedure.

BoT-SORT is an online multi-object tracking method developed to improve identity preservation by combining motion-based association with appearance information and camera-motion compensation [36] as show in Figure 10. For each video frame t, the cabbage detector generates a set of detections using Equation (8).

(8) $$D_{t}=\{d_{t}^{1},\;d_{t}^{2},\ldots ..,d_{t}^{N}\}$$

where each detection contains a bounding box, confidence score, and predicted cabbage class. Existing trajectories are represented by Equation (9).

(9) $$T_{t-1}=\{T_{t-1}^{1},\;T_{t-1}^{2},\ldots ..,T_{t-1}^{M}\}$$

A Kalman-filter-based motion model predicts the expected location of each active trajectory in the current frame. Because the camera itself moves during field acquisition, apparent displacement between consecutive frames can originate from both camera motion and object position. BoT-SORT therefore incorporates camera-motion compensation before data association, reducing the effect of global image displacement on trajectory prediction.

Following motion prediction, detected plants are associated with existing trajectories according to spatial similarity. Intersection over union between a predicted track box B_T_ and a detected box B_D_ is expressed as Equation (10).

(10) $$\mathrm{IoU}(B_{T},B_{D})=\frac{\left|B_{T}\cap B_{D}\right|}{\left|B_{T}\cup B_{D}\right|}$$

The corresponding IoU-based association cost can be expressed as Equation (11).

*C_IoU_* = 1 − IoU(*B_T_*, *B_D_*) (11)

BoT-SORT additionally incorporates appearance information to distinguish detections with similar motion or spatial overlap. Let fT and fD denote appearance feature vectors associated with an existing trajectory and candidate detection, respectively. Their cosine distance can be expressed as Equation (12).

(12) $$C_{\mathrm{app}}=1-\frac{f_{T}^{T}f_{D}}{{\left\|f_{T}\right\|}_{2}{\left\|f_{D}\right\|}_{2}}$$

Baseline tracking used the Ultralytics implementation of BoT-SORT, combining motion prediction, spatial association, appearance information, and sparse optical-flow camera-motion compensation. The high-confidence, low-confidence, and new-track thresholds were 0.25, 0.10, and 0.25, respectively. The track buffer was set to 30 processed frames, and the association matching threshold was 0.80. Detection-score fusion and automatic ReID were enabled, with proximity and appearance settings of 0.50 and 0.80, respectively. These core tracking settings were identical in the baseline and improved configurations. Baseline counting used the original tracker IDs and required at least two observations before an identity became eligible for counting. Tracker and counting states were reset before each video.

#### 2.4.2. Proposed Improved BoT-SORT Algorithm

The improved configuration introduced an additional identity-management layer operating on the baseline BoT-SORT outputs. Each raw tracker ID was mapped to a canonical plant identity. Existing mappings were resolved first, and canonical identities already represented in the current frame were excluded from reassignment. This prevented a newly appearing track fragment from being linked to another identity observed simultaneously.

Newly observed tracker IDs were compared with canonical identities last observed within 12 processed frames. Relinking required agreement between the current detection class and the canonical identity’s majority class. Class consistency was therefore a strict constraint in this additional relinking step; it did not modify the internal BoT-SORT association cost. Candidate positions were predicted from the last centroid and a smoothed velocity estimate, updated as Equation (13).

*v_t_* = 0.65*v_t_*_−1_ + 0.35*v_obs_* (13)

A match was spatially eligible when its distance from the predicted centroid did not exceed 85(1 + 0.04∆t) pixels, where ∆t was the frame gap. Distances were measured in the original video-frame coordinates.

**Figure 11:**
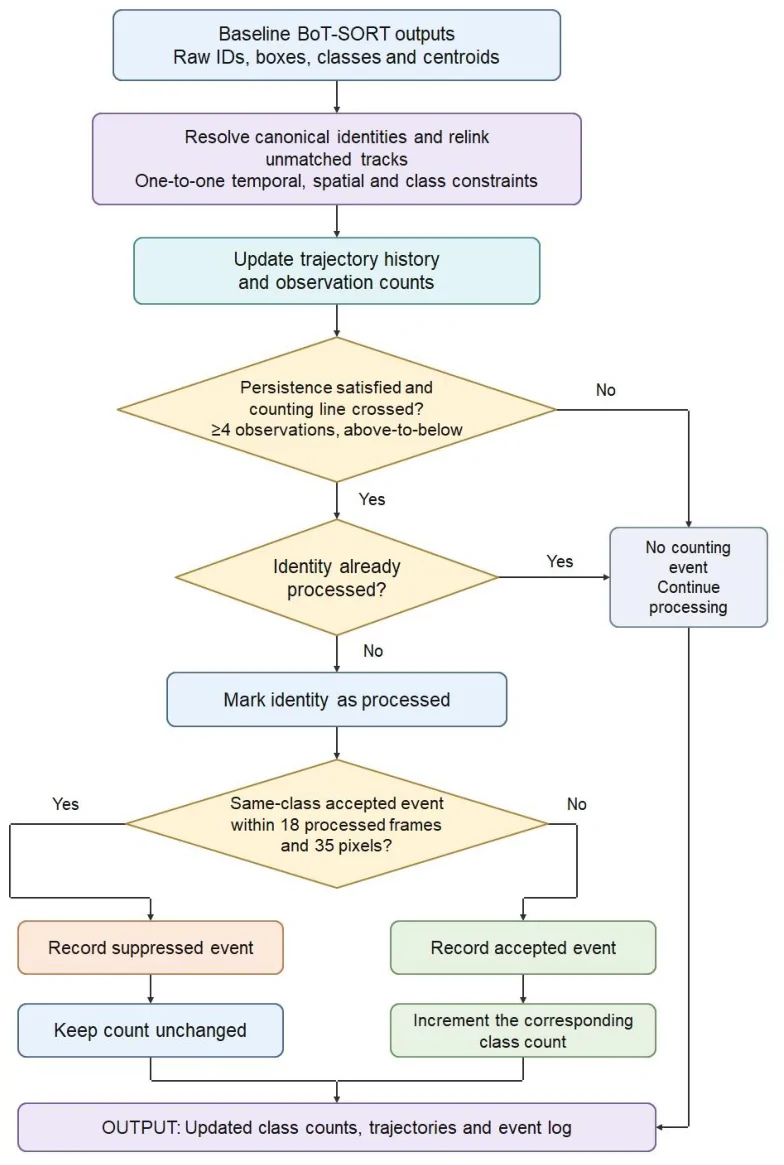
Workflow of the proposed Improved BoT-SORT algorithm for cabbage tracking and cumulative counting.

Eligible correspondences were resolved using one-to-one Hungarian assignment based on spatial distance. Unmatched tracker IDs received new canonical identities, whereas relinked fragments retained the corresponding identity’s observation history, class history, and counting status. Canonical records remained available throughout each video, although relinking a new raw ID was restricted to the specified temporal window. An identity became eligible for counting after observations in at least four distinct processed frames. These observations were cumulative and were not required to be consecutive. The overall improved BoT-SORT workflow is illustrated in Figure 11, and the configuration parameters are mentioned in Table 3. Algorithm 1 shows the pseudocode for the improved BoT-SORT for seedling and cabbage tracking and counting.

Tracking parameters were adopted from an existing implementation, without additional hyperparameter optimization in this study (Table 3). These settings were fixed before final evaluation and applied unchanged across all nine test videos. The test videos were not used for parameter selection or adjustment. Parameter sensitivity and optimality were not evaluated.
**Algorithm 1**. Improved BoT-SORT for plant tracking and countingInput: Video V, detector D, tracker B, and parameters. Output: Plant count N, trajectories T, and event log E. 1:   Reset B and identity mappings; initialize T, E, accepted events A, and processed identities P as empty; N ← 0 2:   for each frame I_t_ in V do 3:         Obtain raw tracks using D and B 4:         Resolve existing identities and reserve them for this frame 5:         Relink unmapped tracks to unreserved identities by one-to-one assignment under temporal, class, and spatial constraints; create new identities for unmatched tracks 6:         Update track states and observation counts; append observations to T 7:         for each observed identity c ∉ P with sufficient observations and a valid above-to-below line crossing do 8:               Add c to P 9:               if duplicate filtering is enabled and a same-class accepted event satisfies the temporal and spatial thresholds then 10:                     Record suppression in E 11:             else 12:                     Add the accepted event to A and E; N ← N + 1 13:             end if 14:         end for 15: end for 16: return N, T, E

#### 2.4.3. Counting Lines and Duplicate Event Filtering

Counting used a horizontal line spanning the full image width at y = 0.60H, where H was the original video height. No additional polygonal region of interest was applied. A crossing candidate was generated when the previous available centroid was above the line and the current centroid was on or below it. Baseline counting used raw tracker IDs, whereas the improved configuration used canonical identities. Crossings occurring before the minimum observation requirement was satisfied were not retrospectively accepted.

In the improved configuration, an eligible crossing candidate was suppressed when an accepted event of the same class occurred within 18 processed frames and within 35 pixels of the candidate’s current centroid. This filter was intended to reduce potentially repeated counting events following identity fragmentation. It was disabled in the baseline configuration.

Processed identities and accepted counting identities were maintained separately. An identity was marked as processed after its eligible crossing candidate was evaluated, whether accepted or suppressed. Only accepted identities increased the plant count. Consequently, the cumulative count equalled the number of accepted identities, and separate counters were maintained for seedlings and grown cabbage plants.

### 2.5. Performance Evaluation

#### 2.5.1. Cabbage Detection

The performance of the baseline YOLOv8 and proposed YOLOv8-CBAM models was evaluated using the same test dataset and evaluation criteria. Detection performance was quantified using precision (P), recall (R), F1-score, average precision (AP), and mean average precision (mAP). Precision and recall were calculated using Equations (14) and (15).

(14) $$P=\frac{\mathrm{TP}}{\mathrm{TP}+\mathrm{FP}}$$

(15) $$R=\frac{\mathrm{TP}}{\mathrm{TP}+\mathrm{FN}}$$

where TP, FP, and FN represent true-positive, false-positive, and false-negative detections, respectively. The F1-score, representing the harmonic mean of precision and recall, was calculated using Equation (16).

(16) $$F1=\frac{2\mathrm{PR}}{P+R}$$

Average precision was obtained from the area under the precision–recall curve using Equation (17).

(17) $$\mathrm{AP}=\int_{0}^{1}P(R)\mathrm{dR}$$

and mAP was calculated across the C target classes using Equation (18).

(18) $$\mathrm{mAP}=\frac{1}{C}\sum_{C=1}^{C}{\mathrm{AP}}_{C}$$

where AP_c_ represents the AP of class c. Detection performance was reported using mAP@0.5 and mAP@0.5:0.95 together with class-wise precision, recall, and F1-score for cabbage seedlings and grown cabbage plants. The contribution of CBAM was evaluated by comparing baseline YOLOv8, channel-attention-only, spatial-attention-only, and complete YOLOv8-CBAM configurations under identical experimental conditions.

#### 2.5.2. Counting Accuracy

The true number of unique cabbage plants in each test video was determined by manual counting and used as the reference for evaluating automated counting performance. The same videos were processed using the four principal detector–tracker combinations. For video i, counting error (CE_i_) was calculated using Equation (19).

CE_i_ = N_a,i_ − N_m,i_ (19)

where N_m,i_ and N_a,i_ represent the manually counted and automatically predicted numbers of plants, respectively. Absolute percentage error (APE) and counting accuracy (CA) were calculated using Equations (20) and (21).

(20) $$\mathrm{APE}=\frac{\left|N_{a,i}-N_{m,i}\right|}{N_{m,i}}\times 100$$

(21) $$\mathrm{CA}=\left(1-\frac{\left|N_{a,i}-N_{m,i}\right|}{N_{m,i}}\right)\times 100$$

The mean absolute percentage error (MAPE) was calculated as the arithmetic mean of the APE values across the nine independent test videos. Overall counting performance across n independent test videos was further quantified using mean absolute error (MAE) and root mean square error (RMSE) using Equations (22) and (23).

(22) $$\mathrm{MAE}=\frac{1}{n}\sum_{i=1}^{n}\left|N_{a,i}-N_{m,i}\right|$$

(23) $$\mathrm{RMSE}=\sqrt{\frac{1}{n}\sum_{i=1}^{n}{\left(\left|N_{a,i}-N_{m,i}\right|\right)}^{2}}$$

Mean absolute percentage error was calculated by averaging the video-level absolute percentage errors, giving equal weight to each of the nine test videos. Mean counting accuracy was calculated as 100 minus this value. The coefficient of determination was calculated directly from the automated and manual counts, without fitting a regression line using Equation (24).

(24) $$R^{2}=1-\frac{\sum_{i=1}^{n}{\left(N_{a,i}-N_{m,i}\right)}^{2}}{\sum_{i=1}^{n}{\left(N_{m,i}-\bar{N_{m}}\right)}^{2}}$$

where $\bar{N_{m}}$ is the mean manual count and n = 9.

#### 2.5.3. Computational Performance

To assess the relative effectiveness of the proposed tracking approach, ByteTrack and DeepSORT were additionally implemented as benchmarks.

Computational efficiency was evaluated using detector inference latency and complete detection–tracking–counting pipeline speed. Detector FPS was calculated using Equation (25).

(25) $${\mathrm{FPS}}_{\det}=\frac{1}{t_{\det}}$$

where t_det_ is the mean detector processing time per frame (s). Complete-pipeline FPS was calculated using Equation (26).

(26) $${\mathrm{FPS}}_{\mathrm{pipeline}}=\frac{N_{f}}{T}$$

where N_f_ is the total number of processed video frames and T is the total processing time. Pipeline timing included detection, trajectory association, identity management, counting, visualization, and result recording. All configurations were evaluated using the same nine test videos and computing platform. Comparisons among configurations were descriptive and based on the observed detection metrics, video-level counting errors, trajectory-continuity indicators, and processing throughput.

#### 2.5.4. Trajectory and Counting-Event Evaluation

Four test videos (V2, V4, V6, and V8) were selected to cover seedling and grown-plant stages in both cultivation environments, without considering algorithm performance. Plants were manually annotated with persistent identities, class labels, and frame-level bounding boxes enclosing their visible extent. Fully occluded plants were not assigned boxes, and their original identities were retained after reappearance when continuity could be established confidently. A second annotator reviewed box placement, labels, identity continuity, and counting events; disagreements were resolved through joint frame inspection.

Tracking performance was evaluated using multiple-object tracking accuracy (MOTA), identity F1 score (IDF1), higher-order tracking accuracy (HOTA), identity switches, and trajectory fragmentations. An IoU threshold of 0.50 was used for MOTA and IDF1, while HOTA used thresholds of 0.05–0.95 at intervals of 0.05. Baseline and improved outputs were evaluated using raw and canonical identities, respectively, under identical annotation rules. Counting eligibility filters were not applied to trajectory evaluation.

For the 1280 × 720-pixel videos, the horizontal counting line extended from (0, 432) to (1279, 432). Each reference plant whose bounding-box center crossed the line from above to below was eligible for one count. Accepted events were checked against the annotated plants: the first event per eligible plant was classified as correct, additional events as duplicates, eligible plants without an event as missed, and events without an eligible reference plant as false counts. Suppressed candidates were inspected separately and were not automatically classified as duplicates.

#### 2.5.5. Statistical Analysis

Detection metrics were summarized as the mean and sample standard deviation across independent training runs. These estimates describe variability associated with training on the fixed data partition. Counting performance was reported separately for each video and through aggregate error metrics. Differences between rounded point estimates were not interpreted as evidence of statistical significance.

## 3. Results

### 3.1. Training and Convergence of the Detection Models

**Figure 12:**
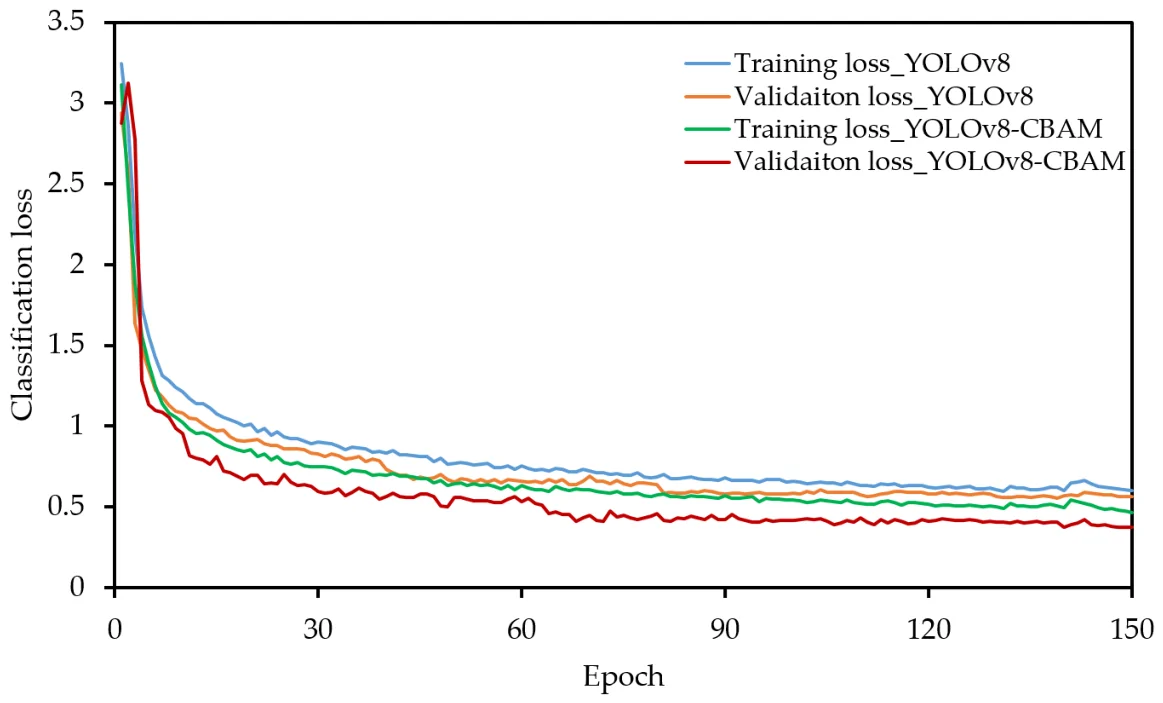
Training and validation loss curves of the baseline YOLOv8 and proposed YOLOv8-CBAM models over 150 epochs using an initial learning rate of 0.001.

The baseline YOLOv8 and proposed YOLOv8-CBAM models were trained for 150 epochs using an initial learning rate of 0.001 under identical training conditions. Figure 12 presents the training and validation classification loss trajectories of the two models. Both architectures exhibited a rapid reduction in loss during the initial training stage, followed by a progressively slower decline and stabilization as training proceeded. This trend indicates effective optimization and convergence of both detection models.

During the early epochs, relatively large changes in loss were observed as the models rapidly learned discriminative representations from the training data. After approximately 20–30 epochs, the loss curves decreased more gradually, with only minor fluctuations during the remaining training period. Importantly, the validation losses generally followed the corresponding training-loss trends throughout training, without a pronounced sustained increase during the later epochs. The relatively small separation between the training and validation curves therefore indicates stable generalization without clear evidence of severe overfitting under the adopted training configuration.

The YOLOv8-CBAM model exhibited a lower loss trajectory than the baseline YOLOv8 over much of the training process, particularly during the middle and later epochs. At the end of training, both the training and validation losses of YOLOv8-CBAM were lower than those of the baseline model. This behavior suggests that incorporating channel and spatial attention into the selected feature-fusion locations facilitated more effective feature refinement during optimization. Nevertheless, loss values alone were not used to determine model superiority; the final models were further evaluated using precision, recall, mAP@0.50, and mAP@0.50:0.95, as presented in Section 3.2.

### 3.2. Detection Performance of YOLOv8 and YOLOv8-CBAM

The detection performance of YOLOv8 and YOLOv8-CBAM was evaluated using precision, recall, mAP@0.50, and mAP@0.50:0.95, as shown in Table 4. In addition to overall test-set performance, the models were evaluated separately for greenhouse seedlings, greenhouse-grown cabbage, open-field seedlings, and open-field-grown cabbage to examine their performance across differences in plant size, canopy development, background characteristics, and cultivation environment.

The magnitude of improvement varied with cultivation environment and growth stage. The largest improvement in localization performance occurred for greenhouse seedlings, where mAP@0.50:0.95 increased from 0.53 to 0.59, while precision increased from 0.82 to 0.88. For greenhouse-grown cabbage, YOLOv8-CBAM increased mAP@0.50 from 0.92 to 0.94 and mAP@0.50:0.95 from 0.55 to 0.61. However, recall decreased slightly from 0.91 to 0.89, indicating that the improvement was not uniform across all evaluation metrics.

Under open-field conditions, YOLOv8-CBAM also produced higher mAP values for both growth stages. For seedlings, mAP@0.50 increased from 0.93 to 0.96, while mAP@0.50:0.95 increased from 0.59 to 0.62. For grown cabbage, the corresponding values increased from 0.92 to 0.95 and from 0.58 to 0.62, respectively. Although precision for open-field-grown cabbage decreased from 0.90 to 0.87, recall increased from 0.89 to 0.93. This indicates a shift toward detecting a greater proportion of plants at the expense of a modest increase in false-positive detections. Nevertheless, the simultaneous increases in both mAP measures indicate improved overall detection performance.

**Figure 13:**
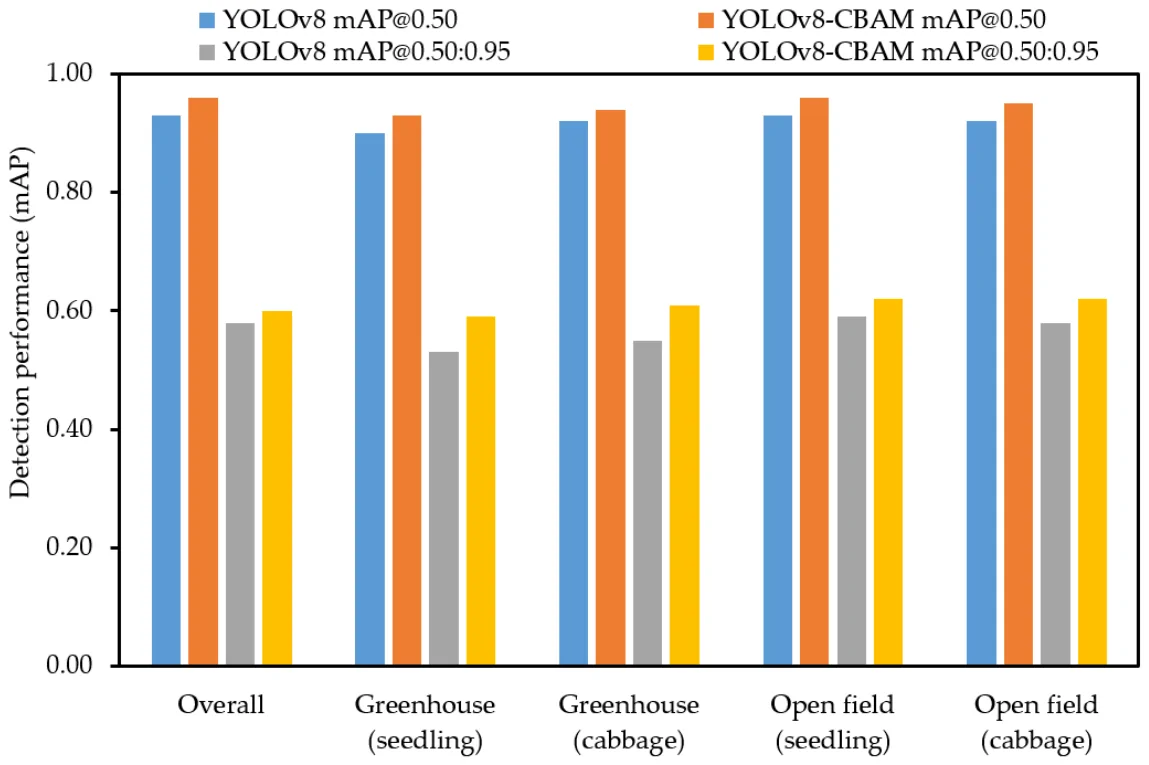
Comparison of mAP@0.50 and mAP@0.50:0.95 between YOLOv8 and YOLOv8-CBAM under greenhouse and open-field conditions for seedling and grown-cabbage detection.

The condition-specific results are summarized visually in Figure 13. Across all four cultivation and growth-stage conditions, YOLOv8-CBAM produced higher mAP@0.50 and mAP@0.50:0.95 values than baseline YOLOv8. The improvement was particularly evident for the stricter mAP@0.50:0.95 metric in the greenhouse datasets, where increases of 0.06 were obtained for both seedlings and grown cabbage. These results indicate that the attention-enhanced model provided more consistent localization across IoU thresholds under the evaluated conditions. Both cultivation environments were represented in detector training. Therefore, the condition-specific results describe performance within the sampled environments and do not establish transferability to independent sites or seasons.

**Figure 14:**
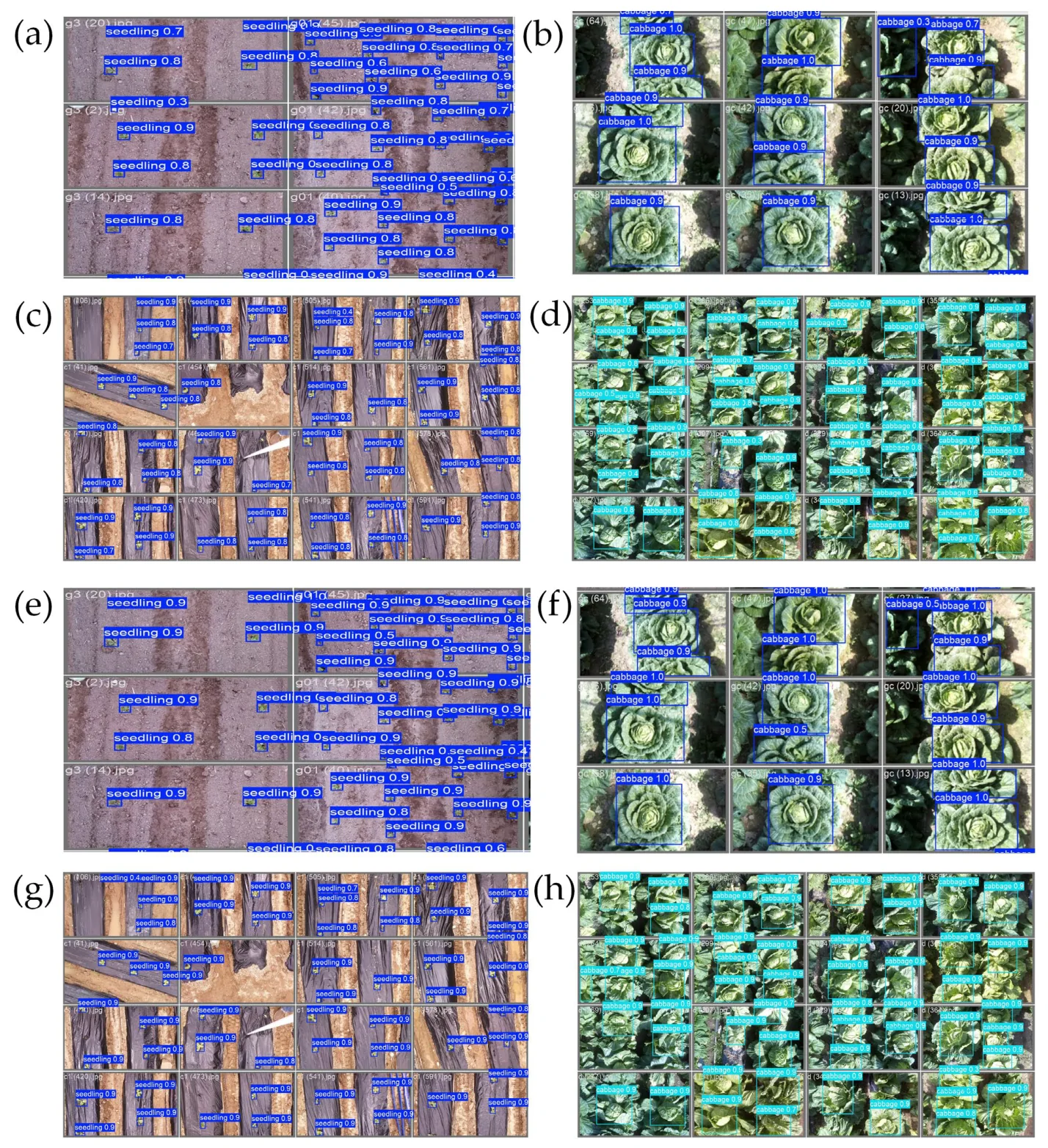
Representative detection outputs of YOLOv8 model for (**a**) greenhouse seedlings, (**b**) greenhouse-grown cabbage, (**c**) open-field seedlings, and (**d**) open-field-grown cabbage, and YOLOv8-CBAM for (**e**) greenhouse seedlings, (**f**) greenhouse-grown cabbage, (**g**) open-field seedlings, and (**h**) open-field-grown cabbage.

Representative detection outputs are presented in Figure 14 for greenhouse seedlings, greenhouse-grown cabbage, open-field seedlings, and open-field-grown cabbage. The examples illustrate the ability of both models to detect cabbage plants across substantial differences in target size, plant spacing, canopy development, soil/background appearance, and plant overlap. The YOLOv8-CBAM outputs qualitatively support the quantitative results by maintaining plant detection across both small seedling targets and larger developed canopies.

### 3.3. Ablation of CBAM

An ablation analysis was conducted to quantify the individual and combined contributions of channel attention (CA) and spatial attention (SA) to the proposed YOLOv8-CBAM detector. Four configurations were evaluated under identical training and testing conditions: baseline YOLOv8, YOLOv8 with CA only, YOLOv8 with SA only, and the complete YOLOv8-CBAM model incorporating sequential channel and spatial attention. For all attention-based configurations, the attention modules were introduced at the same selected feature-fusion locations in the YOLOv8 neck.

As shown in Table 5, the baseline YOLOv8 achieved a precision of 0.85, recall of 0.90, mAP@0.50 of 0.93, and mAP@0.50:0.95 of 0.58. Introducing CA alone increased precision and mAP@0.50 to 0.87 and 0.94, respectively, although recall decreased slightly to 0.89 and mAP@0.50:0.95 to 0.57. The SA-only configuration produced a higher recall of 0.91 and mAP@0.50 of 0.95, but its mAP@0.50:0.95 similarly remained at 0.57. The complete YOLOv8-CBAM configuration provided the best overall detection performance, achieving 0.88 precision, 0.92 recall, 0.96 mAP@0.50, and 0.60 mAP@0.50:0.95. Relative to baseline YOLOv8, these correspond to absolute improvements of 0.03, 0.02, 0.03, and 0.02, respectively. The results indicate that CA or SA alone improved selected aspects of detection performance, whereas their sequential combination provided a more balanced improvement across precision, recall, and both mAP measures.

**Figure 15:**
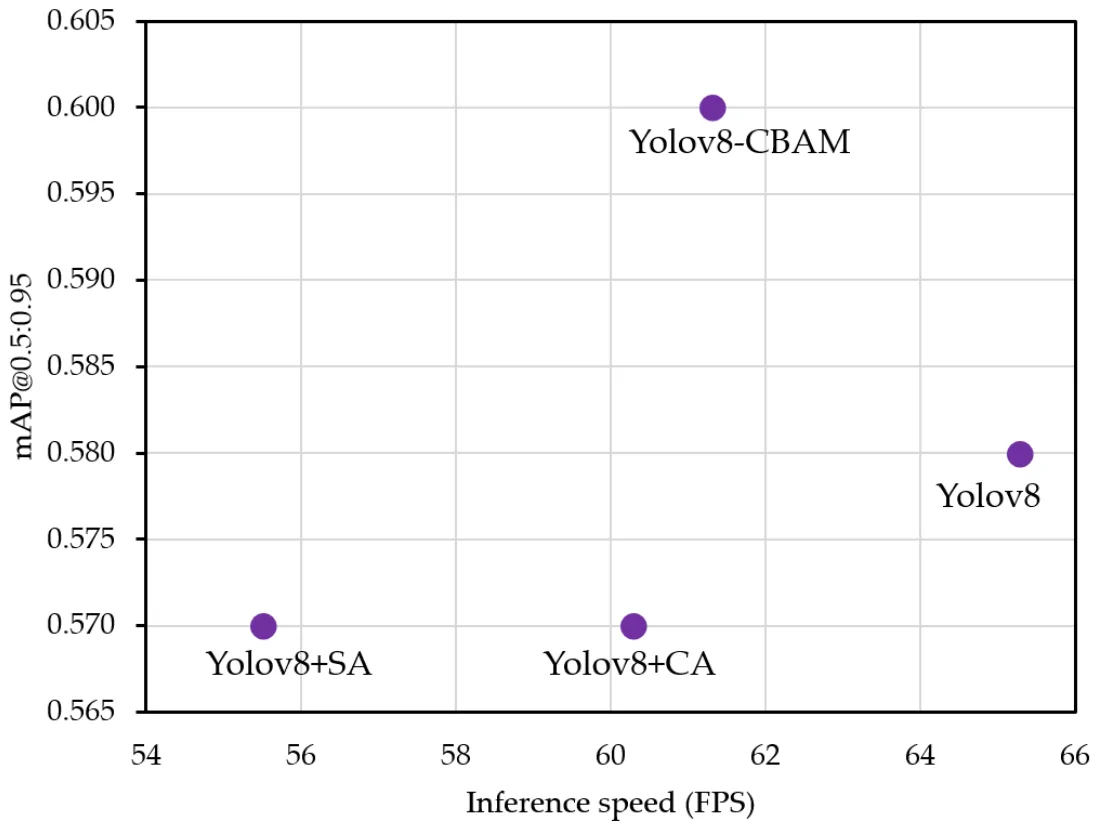
Accuracy–speed trade-off obtained from the CBAM ablation analysis.

The detector speed decreased from 65.3 to 61.3 FPS (approximately 6.1%). In comparison, the SA-only model achieved only 55.6 FPS, despite its lower mAP@0.50:0.95 of 0.57. As illustrated in Figure 15, the complete YOLOv8-CBAM configuration achieved the highest mAP@0.50:0.95 (0.60) while maintaining a detector processing speed of 61.3 FPS, demonstrating a favorable balance between detection accuracy and computational efficiency compared with the baseline, CA-only, and SA-only configurations.

The computational complexity and storage requirements of the evaluated detectors are shown in Appendix A Table A4. YOLOv8n–CBAM contained 3.08 million parameters, compared with 3.01 million for the baseline, representing an increase of approximately 2.3%. Its model size increased from 6.50 to 6.95 MB. The channel-attention and spatial-attention variants contained 3.04 and 3.03 million parameters, respectively. The computational costs ranged around 8.20 GFLOPs. These measurements indicate a modest increase in parameter count and storage requirements for the complete CBAM configuration.

Overall, the ablation results demonstrate that the performance improvement of the proposed detector cannot be attributed solely to either channel or spatial attention. Instead, sequential channel–spatial feature refinement produced the highest overall detection performance. Based on these results, the complete YOLOv8-CBAM architecture was selected for the subsequent tracking and cabbage-counting experiments.

### 3.4. Cabbage Counting Accuracy

Cabbage counting performance was evaluated across nine independent test videos by comparing the automatically estimated plant populations with manually verified reference counts. The reference counts ranged from 22 to 81 plants per video. The four detector–tracker configurations were evaluated using counting error (CE), mean absolute percentage error (MAPE), counting accuracy, mean absolute error (MAE), root mean square error (RMSE), and coefficient of determination (R^2^).

As summarized in Table 6, the baseline YOLOv8–BoT-SORT configuration achieved a counting accuracy of 93.34%, with an MAE of 3.00 plants, RMSE of 3.35 plants, and R^2^ of 0.962. Incorporating Improved BoT-SORT increased the counting accuracy to 94.77% and reduced the MAE and RMSE to 2.33 and 2.65 plants, respectively. The corresponding R^2^ increased to 0.976, indicating improved agreement between the automated and manual counts.

Using the YOLOv8-CBAM detector, baseline BoT-SORT achieved a mean counting accuracy of 96.84%, an MAE of 2.22 plants, an RMSE of 2.49 plants, and an R^2^ of 0.979. The improved BoT-SORT configuration achieved a mean counting accuracy of 99.27%, an MAE of 0.56 plants, an RMSE of 0.88 plants, and an R^2^ of 0.997. Relative to baseline BoT-SORT, the improved configuration increased counting accuracy by 2.43% and reduced MAE and RMSE by 75.0% and 64.6%, respectively.

The negative mean counting errors indicate an overall tendency toward undercounting across all six configurations. The magnitude of this bias decreased from −3.00 plants for YOLOv8–BoT-SORT to −0.56 plants for YOLOv8-CBAM–Improved BoT-SORT. Relative to the baseline configuration, the complete framework increased mean counting accuracy from 93.34% to 99.27%, an improvement of 5.93 percentage points. MAE decreased from 3.00 to 0.56 plants, while RMSE decreased from 3.35 to 0.88 plants. These results indicate that the combined detector and tracker modifications produced the lowest observed counting errors among the evaluated configurations.

Using the same YOLOv8-CBAM detector, replacing baseline BoT-SORT with Improved BoT-SORT increased counting accuracy from 96.84% to 99.27% and reduced mean APE from 3.16% to 0.73%. The corresponding decreases in MAE and RMSE indicate that the modified tracking and counting procedure provided an additional improvement beyond that obtained through detector modification alone.

At the individual-video level, the complete framework exactly reproduced the manually verified counts in five of the nine videos: V1, V3, V4, V6, and V7. It underestimated V2, V5, and V9 by one plant each and V8 by two plants. Together with an R^2^ of 0.997, these results demonstrate close agreement between automated and manual counts under the evaluated conditions. Agreement in cumulative counts does not exclude compensating missed and duplicate events. These error sources were evaluated separately on four annotated videos, as reported in Section 3.6.

### 3.5. Comparison with ByteTrack and DeepSORT

ByteTrack, DeepSORT, baseline BoT-SORT, and Improved BoT-SORT were evaluated using the same YOLOv8-CBAM detector and nine test videos. Manually verified counts ranged from 22 to 81 plants per video. Counting performance was compared using mean counting accuracy, MAE, RMSE, and R^2^.

As shown in Table 7, ByteTrack achieved a counting accuracy of 94.45%, an MAE of 3.33 plants, an RMSE of 3.50 plants, and an R^2^ of 0.959. DeepSORT achieved a counting accuracy of 96.03%, whereas baseline BoT-SORT achieved 96.84%. Both methods produced an MAE of 2.22 plants, an RMSE of 2.49 plants, and an R^2^ of 0.979. Their different percentage accuracies reflect differences in the distribution of errors among videos with different reference counts.

Improved BoT-SORT achieved the highest observed counting accuracy of 99.27%, with an MAE of 0.56 plants, an RMSE of 0.88 plants, and an R^2^ of 0.997. It increased counting accuracy by 4.82, 3.24, and 2.43 percentage points relative to ByteTrack, DeepSORT, and baseline BoT-SORT, respectively. MAE was reduced by 83.3% relative to ByteTrack and by 75.0% relative to both DeepSORT and baseline BoT-SORT.

The improved configuration produced exact counts in five of nine videos, while the remaining errors were undercounts of one or two plants. These descriptive comparisons support improved cumulative counting performance under the evaluated conditions. These counting comparisons do not independently establish trajectory correctness; direct tracking and counting-event evaluation of the two BoT-SORT configurations is presented in Section 3.6.

### 3.6. Performance Comparison of Tracking Algorithm

Tracking and counting-event performance was evaluated on four annotated videos (V2, V4, V6, and V8), covering seedling and grown-plant stages in both cultivation environments. With the YOLOv8-CBAM detector held constant, Improved BoT-SORT achieved mean MOTA, IDF1, and HOTA values of 88.4%, 82.3%, and 83.2%, respectively, exceeding baseline BoT-SORT by 6.2, 2.7, and 8.9 percentage points, as shown in Table 8. Total identity switches decreased from 23 to 12, while trajectory fragmentations decreased from eight to three.

Event-level evaluation identified 11 missed plants with baseline BoT-SORT and three with Improved BoT-SORT, with no duplicate or false counts observed for either configuration. Consequently, the predicted totals were 275 and 283 plants, respectively, compared with 286 manually counted plants, consistent with the Appendix A Table A3 per-video results. These findings indicate improved identity continuity and fewer missed plants on the evaluated subset. However, they do not demonstrate a reduction in duplicate counts, because neither configuration produced duplicate events on these videos, and the contributions of individual tracking modifications were not isolated.

### 3.7. Computational Performance and Field Examples

The computational performance of the four principal detector–tracker configurations was evaluated across the nine test videos using the complete detection–tracking–counting pipeline. The reported processing speed therefore included plant detection, trajectory association, identity management, counting operations, visualization, and result recording. As summarized in Table 9, YOLOv8–BoT-SORT achieved the highest mean pipeline processing speed among the four principal configurations at 24.33 FPS, followed by YOLOv8-CBAM–BoT-SORT (23.42 FPS) and YOLOv8-CBAM–Improved BoT-SORT (23.38 FPS). YOLOv8–Improved BoT-SORT operated at 21.14 FPS.

For the final YOLOv8-CBAM–Improved BoT-SORT configuration, the mean detector latency was 16.31 ms frame^−1^, corresponding to a detector-only processing rate of approximately 61.3 FPS, whereas the complete detection–tracking–counting pipeline operated at 23.38 FPS. The difference between detector-only and complete-pipeline processing rates reflects the additional computation required for trajectory association, identity management, counting logic, visualization, and output recording.

The complete pipeline throughput of 23.38 FPS was below the 30-FPS acquisition rate and therefore did not establish continuous processing of every incoming frame without buffering, frame reduction, or further optimization. These results indicate near-real-time processing on the evaluated RTX 3090 workstation. Processing on the portable acquisition device was not assessed.

Adding the improved tracking procedure increased mean counting accuracy from 96.84% to 99.27%, a gain of 2.43 percentage points, while mean pipeline throughput changed from 23.42 to 23.38 FPS. Thus, the complete configuration achieved higher counting accuracy with a small observed difference in processing speed under the evaluated conditions. Because repeated timing variability was not quantified, the throughput difference should be interpreted descriptively.

## 4. Discussion

The present study demonstrated that combining attention-enhanced object detection with trajectory-aware multi-object tracking can provide accurate video-based counting of cabbage seedlings and grown cabbage plants under both greenhouse and open-field cultivation conditions. The subsequent improvement of BoT-SORT further increased cumulative counting accuracy through trajectory-persistence and duplicate-suppression mechanisms intended to reduce trajectory fragmentation and repeated counting. This distinction between detection and counting is important because accurate frame-wise plant recognition does not necessarily translate directly into accurate population estimates from continuous video. Previous studies have demonstrated the suitability of deep-learning detectors for crop and seedling identification [14,15,16,17,23,25,26,27], but practical video-based crop monitoring additionally requires temporal association of detections so that the same plant is not counted repeatedly as the camera moves through the field.

Table 10 compares the present framework with related cabbage detection and counting studies. Chen et al. [10] and Zhao et al. [14] investigated UAV-based seedling monitoring, whereas Jiang et al. [25] focused on field cabbage detection and three-dimensional positioning. The present study evaluates portable RGB video counting across seedling and grown-plant stages under both sampled cultivation environments. Differences in datasets, imaging conditions, metric definitions, and processing hardware preclude direct performance ranking; the comparison instead highlights the scope and evaluation approach of each framework.

Detection and counting metrics are reproduced using the definitions reported in each study. Dataset sizes refer to images before augmentation where this information is available. Inference times and pipeline throughput differ in processing scope and hardware and should not be directly ranked. The present study’s mean counting accuracy was calculated as 100% minus mean absolute percentage error across nine videos.

The baseline YOLOv8 model already provided relatively high detection performance, with overall precision, recall, mAP@0.50, and mAP@0.50:0.95 values of 0.85, 0.90, 0.93, and 0.58, respectively. Incorporation of CBAM increased these values to 0.88, 0.92, 0.96, and 0.60. More importantly, YOLOv8-CBAM improved mAP under all four combinations of cultivation environment and crop growth stage evaluated in this study. The improvement was particularly evident for greenhouse seedlings, where mAP@0.50:0.95 increased from 0.53 to 0.59, and for greenhouse-grown cabbage, where it increased from 0.55 to 0.61. These results indicate that the attention mechanism improved not merely detection at a relatively permissive IoU threshold, but also localization consistency across a broader range of IoU thresholds.

This improvement is consistent with the challenges reported in previous agricultural object-detection studies. Seedlings frequently represent small targets with relatively limited discriminative visual information, whereas grown cabbage plants develop large, irregular and overlapping leaf canopies. Previous studies have therefore modified YOLO architectures or their feature-fusion mechanisms to improve detection of cabbage heads, seedlings and other agricultural targets under complex backgrounds [15,16,17,18,19,20,23,25,26,27]. Jiang et al. [25] investigated cabbage detection and positioning using an improved YOLOv8n model, Zhao et al. [14] evaluated early-stage cabbage seedling detection and counting from UAV RGB imagery, and Li et al. [22] examined rice-seedling density estimation using an improved YOLOv8 model. Collectively, these studies indicate that crop scale, canopy structure, occlusion and background variation remain major determinants of detection performance. The present results extend this concept by demonstrating that a single detector can accommodate both the relatively small appearance of transplanted seedlings and the substantially larger canopy structure of grown cabbage across two cultivation environments.

The ablation experiment compared the individual and combined contributions of channel and spatial attention under the evaluated conditions. Channel attention alone increased precision from 0.85 to 0.87 and mAP@0.50 from 0.93 to 0.94, whereas spatial attention alone increased recall to 0.91 and mAP@0.50 to 0.95. However, neither individual component improved mAP@0.50:0.95 above the baseline value of 0.58. In contrast, sequential channel and spatial attention produced the strongest overall performance, with precision of 0.88, recall of 0.92, mAP@0.50 of 0.96, and mAP@0.50:0.95 of 0.60. This result is consistent with the conceptual operation of CBAM [39], in which channel attention emphasizes informative feature channels and spatial attention subsequently emphasizes relevant locations within those features. For cabbage imagery, this complementary mechanism is particularly appropriate because successful recognition requires both discrimination of plant-related visual characteristics and localization of those characteristics against soil, plastic mulch, shadows and overlapping vegetation.

The ablation results suggest that combining channel and spatial attention improved detection performance on the evaluated cabbage dataset compared with either component alone. Although the individual components improved selected metrics, neither increased mAP@0.50:0.95 above the baseline value of 0.58, whereas the complete CBAM configuration achieved 0.60. This finding is consistent with the complementary roles of channel attention in weighting feature channels and spatial attention in emphasizing informative regions [39]. These results describe variability across five independent training runs on the fixed dataset partition and do not establish transferability to independent sites or seasons. Practical suitability should also be assessed using detector latency and complete-pipeline throughput alongside detection accuracy and counting error, because detector-only speed does not represent the processing requirements of the entire monitoring framework.

Differences among cultivation conditions also provide insight into the behavior of the proposed detector. The greatest improvement in the stricter mAP@0.50:0.95 metric occurred for the greenhouse datasets, where increases of 0.06 were obtained for both seedlings and grown cabbage. In greenhouse-grown cabbage, however, recall decreased slightly from 0.91 to 0.89 despite improvements in precision and both mAP measures. Conversely, for open-field-grown cabbage, precision decreased from 0.90 to 0.87 while recall increased from 0.89 to 0.93. These opposing changes indicate that attention enhancement altered the balance between false-positive and false-negative detections depending on the visual environment. Such variation is reasonable because illumination, background texture, leaf overlap and canopy density differ considerably between greenhouse and open-field cultivation. Cabbage-specific studies have examined detection under variable field illumination and natural backgrounds [25], head localization under different occlusion levels [13], and the effect of light occlusion on online recognition [26]. These studies provide context for the visual challenges considered here, whereas soybean and rice investigations concern related but distinct crop applications [21,22]. Thus, evaluating only pooled model performance would have obscured environmentally dependent behavior that is relevant to practical deployment.

The tracking results further demonstrate why detector accuracy alone is insufficient for video-based plant counting. Agricultural plants are essentially stationary targets while the imaging platform moves, meaning that the apparent movement of objects across consecutive frames is largely generated by camera motion. Temporary occlusion, motion blur or missed detections can interrupt a trajectory and cause the same physical plant to receive a new identity when it reappears. This issue is particularly important for crop rows because adjacent plants often have similar color, morphology and appearance. DeepSORT uses appearance descriptors to assist identity association [9,30,31], whereas ByteTrack retains lower-confidence detections to improve association continuity [32]. BoT-SORT additionally incorporates motion and appearance information with camera-motion compensation [36], making it conceptually suitable for the moving-camera configuration used in this study.

The counting results indicate that the modified tracking–counting procedure improved population estimates when used with the same YOLOv8-CBAM detector. Replacing baseline BoT-SORT with Improved BoT-SORT increased mean counting accuracy from 96.84% to 99.27%, while MAE decreased from 2.22 to 0.56 plants and RMSE decreased from 2.49 to 0.88 plants. Final counting accuracy and identity continuity were evaluated separately because missed plants and duplicate events can offset each other in the final count. On the four annotated videos, Improved BoT-SORT increased mean IDF1 from 79.6% to 82.3% and mean HOTA from 74.3% to 83.2%, while reducing identity switches from 23 to 12 and trajectory fragmentations from eight to three (Table 8). These findings support improved identity continuity on the evaluated subset. Event-level analysis also showed fewer missed plants, decreasing from 11 to three. Neither configuration produced duplicate or false counts; therefore, these results do not demonstrate a reduction in duplicate counting. The observed improvements reflect the combined tracking modifications and cannot be attributed to individual components without further ablation experiments.

The comparison with alternative trackers showed counting accuracies of 94.45% for ByteTrack and 96.03% for DeepSORT, compared with 99.27% for Improved BoT-SORT. The improved configuration also produced the lowest observed MAE and RMSE among the evaluated methods. These findings indicate that tracker selection and counting rules can influence final population estimates even when the detector remains unchanged. Event-level evaluation was restricted to the two BoT-SORT configurations on four videos; corresponding trajectory and event-level comparisons with ByteTrack and DeepSORT remain unavailable. The superior performance of the BoT-SORT-based approaches is consistent with the importance of robust association and camera-motion handling in moving-camera applications [30,32,36]. Previous agricultural studies have successfully integrated detection and tracking for crop monitoring and counting [16,20,33,34,35,37,38]; however, the present findings further demonstrate that tracker selection and trajectory-management rules can substantially influence final plant-count estimates even when the detector remains unchanged.

The complete detector–tracker evaluation also demonstrates the complementary contributions of the two proposed modifications. The six detector–tracker configurations showed progressively higher mean counting accuracy, from 93.34% for YOLOv8–BoT-SORT to 94.77% for YOLOv8–Improved BoT-SORT, 96.84% for YOLOv8-CBAM–BoT-SORT, and 99.27% for the complete framework. Between the baseline and complete configurations, MAE decreased from 3.00 to 0.56 plants, and RMSE decreased from 3.35 to 0.88 plants. This pattern is consistent with complementary contributions from detector modification and tracking–counting refinement. However, the aggregate metrics do not directly establish which individual error mechanisms were responsible for the observed improvements.

The remaining counting error was small but showed a systematic direction. The complete framework produced exact counts or slight undercounts across the nine videos, with a mean signed error of −0.56 plants. Exact agreement occurred in V1, V3, V4, V6, and V7. V2, V5, and V9 were underestimated by one plant each, while V8 was underestimated by two plants. Potential causes include temporary occlusion, missed detections, or valid trajectories failing the persistence or crossing requirements. This trade-off is partly inherent to conservative duplicate-suppression rules: stricter trajectory validation reduces repeated counting but may reject short valid trajectories. Similar challenges have been reported in automated plant-counting studies where occlusion, small target size, image quality and dense crop distributions affect the correspondence between detected and actual plant populations [6,16,23,25,26,27]. Consequently, future optimization should consider the balance between suppressing duplicate identities and retaining short but legitimate plant trajectories.

From a practical perspective, the final framework maintained a useful balance between accuracy and computational demand. The YOLOv8-CBAM–Improved BoT-SORT pipeline processed video at 23.38 FPS, compared with 23.42 FPS for YOLOv8-CBAM with baseline BoT-SORT. The complete configuration increased mean counting accuracy by 2.43 percentage points, while observed throughput changed from 23.42 to 23.38 FPS. Because timing variability was not quantified, this small speed difference should be interpreted descriptively. The detector alone operated at approximately 61.3 FPS, whereas the complete pipeline operated at 23.38 FPS because trajectory association, identity management, visualization and counting required additional computation. Although this rate remained below the original 30-FPS acquisition rate, it can reasonably be characterized as near-real-time rather than strictly real-time processing under the evaluated hardware conditions. Previous agricultural YOLO applications similarly emphasize the need to balance inference speed with accuracy for practical field monitoring [15,17,19,23,26].

A further strength of the study is that detection was evaluated across two substantially different growth stages and two cultivation environments rather than under a single crop condition. Previous cabbage research has commonly concentrated either on early seedling emergence and stand assessment [10,14] or on mature cabbage recognition, positioning and harvesting-related applications [13,25,26]. The present framework bridges these stages by using the same overall detection and tracking architecture for both seedlings and grown plants. This capability is agronomically relevant because plant population information has different uses throughout the production cycle: early counts can support stand-establishment assessment and identification of missing plants, whereas later monitoring can contribute to crop-population verification, spatial crop assessment and harvest planning [6,8,11,12]. The use of a relatively low-cost RGB imaging platform further supports the potential for practical adoption compared with monitoring systems that depend on specialized sensing hardware.

Several limitations should nevertheless be considered when interpreting these results. First, although acquisition-group partitioning reduced overlap between dataset subsets, the dataset covered a limited geographical and temporal range. Independent-site and independent-season evaluations are needed before broader applicability can be established. Performance under other cultivars, seasons, soil backgrounds, planting geometries, illumination extremes and weather conditions therefore remains to be established. Second, the nine test videos provided useful independent evaluation of counting performance but remain insufficient to characterize the full range of field variability expected in commercial deployment. Third, trajectory and counting-event evaluation was restricted to four annotated videos, limiting the scope of conclusions about identity management. In addition, no tracking-component ablation was performed. The individual contributions and necessity of minimum trajectory persistence, short-gap relinking, class consistency, and duplicate-event filtering therefore remain unestablished. Finally, the complete pipeline was evaluated on the current computing platform rather than a resource-constrained embedded device, so additional optimization and model compression may be required for fully autonomous deployment. Robustness to image degradation remains a further research direction. Gu and Chen [43] investigated degradation-aware distillation for indoor detection under blur, noise, and compression, with auxiliary components removed before inference. Its application to cabbage monitoring would require separate evaluation under agricultural acquisition conditions. Cultivation environment was confounded with acquisition site, year, season, and potentially crop-management conditions. Consequently, performance differences between greenhouse and open-field subsets cannot be attributed solely to cultivation environment. Evaluation at independent sites and seasons is needed to assess transferability.

Overall, the results demonstrate that reliable cabbage counting from continuous RGB video requires coordinated optimization of detection, temporal identity preservation and counting logic, rather than improvement of frame-wise detection alone. The attention-enhanced YOLOv8 detector improved plant representation across growth stages and cultivation environments, while the improved BoT-SORT procedure converted these detections into trajectory-based counts with substantially reduced cumulative counting error. The resulting 99.27% counting accuracy and R^2^ of 0.997, together with near-real-time processing at 23.38 FPS, indicate that the framework has potential for automated stand assessment and crop-population monitoring. Future work should extend validation across additional farms, seasons and cabbage cultivars and investigate deployment on edge devices or mobile agricultural platforms. Integration with GPS, visual odometry or row-level localization could further convert individual plant trajectories into georeferenced population maps, allowing detected plant counts and missing-plant locations to be incorporated directly into precision crop-management systems.

## 5. Conclusions

This study developed and evaluated an RGB image- and video-based framework combining YOLOv8n-CBAM with Improved BoT-SORT for detecting and counting cabbage seedlings and grown plants under sampled greenhouse and open-field conditions. The framework integrated attention-enhanced detection with trajectory association, identity management, and counting-event validation. Detection, tracking, counting, and computational performance were assessed separately to distinguish the outcomes supported by each evaluation.

The YOLOv8n-CBAM detector achieved a precision of 0.88, recall of 0.92, mAP@0.50 of 0.96, and mAP@0.50:0.95 of 0.60. The attention ablation showed that the combined channel and spatial attention configuration achieved the highest observed mAP@0.50:0.95, although the individual attention components did not improve this metric over the baseline. Across nine test videos, replacing baseline BoT-SORT with the complete Improved BoT-SORT procedure, while retaining the YOLOv8n-CBAM detector, increased mean counting accuracy from 96.84% to 99.27% and reduced MAE from 2.22 to 0.56 plants. The complete configuration achieved an RMSE of 0.88 plants and an R^2^ of 0.997.

Trajectory evaluation on four annotated videos provided additional evidence beyond agreement in final counts. Mean IDF1 increased from 79.6% to 82.3%, mean HOTA increased from 74.3% to 83.2%, and total identity switches decreased from 23 to 12. These results support improved identity association within the annotated subset. Nevertheless, exact counts were obtained in five of the nine videos, with residual undercounting in the remaining four. Complete-pipeline throughput reached 23.38 FPS on the RTX 3090 workstation, indicating near-real-time processing under the evaluated settings, rather than demonstrated continuous processing at the 30 FPS acquisition rate.

The findings indicate potential for cabbage population monitoring within the tested conditions. Broader applicability remains limited by the sampled sites and acquisition periods, environmental confounding, and the restricted trajectory-annotation subset. Because the tracking modifications were evaluated jointly, their individual contributions and necessity remain unestablished. Future work should prioritize independent-site and independent-season validation, tracking-component ablation, analysis of residual counting failures, and testing on the intended deployment hardware.

## Figures and Tables

**Table 1:** Specification of the microcontroller and the camera used in this study.

Microcontroller	RGB Camera
Model: Raspberry Pi 4B board CPU: Quad core Cortex-A72, 64-bit@1.5GHz RAM: 8GB LPDDR4-3200 Connection: 802.11ac wireless, Bluetooth 5.0, BLE, Gigabit Ethernet, 40 pin GPIO header OpenGL ES 3.0 graphics Micro-SD card slot: 32 GB; operating system and data storage Power: 5V DC; USB-C connector and GPIO Operating temp range: 0 to 50 °C	Model: Raspberry Pi camera V2 Image sensor: Sony IMX 219 PQ CMOS Sensor size: 3.68 mm × 2.76 mm Lens size: 1/4″ Resolution: 8 MP Image resolution: 3280 × 2464 pixel Video resolution: 1280 × 720 pixel Pixel size: 1.12 × 1.12 µm Video/Image mode: 1080p: 30fps; 720p: 60fps Image control: Automatic Connection: 15-pin MIPI CSI-2

**Table 2:** Training parameters and computing environment used for the baseline YOLOv8 and proposed YOLOv8-CBAM models.

Category	Parameter	Setting
Model	Baseline model	YOLOv8n
	Proposed model	YOLOv8-CBAM
Training	Number of classes	2
	Classes	seedling, cabbage
	Weight initialization	COCO-pretrained weights
	Input image size	640 × 640 pixels
	Epochs	150
	Batch size	16
	Optimizer	SGD
	Initial learning rate	0.001
	Momentum	0.937
	Weight decay	0.0005
	Early-stopping patience	50 epochs
	Random seed	42 to 46
Augmentation	Horizontal flipping	Enabled
	Vertical flipping	Enabled
	Shift	Enabled
	Zoom	Enabled
	Rotation	Limited
Inference	Confidence threshold	0.25
	IoU threshold	0.70
Software	Programming language	Python 3.12
	Deep-learning framework	PyTorch 2.5.1
	Detection framework	Ultralytics 8.3
Hardware	GPU acceleration	CUDA 12.1
	Operating system	Windows 11, 64-bit
	CPU	Intel Core i9-10900K, 3.70 GHz
	System memory	128 GB
	GPU	NVIDIA GeForce RTX 3090
	GPU memory	24.0 GB

**Table 3:** Configuration parameters and counting rules for baseline BoT-SORT and Improved BoT-SORT.

Setting	Baseline Configuration	Improved Configuration
Detector confidence/NMS IoU	0.25/0.70	Same
Tracking high/low/new thresholds	0.25/0.10/0.25	Same
Track buffer	30 processed frames	Same
Association matching setting	0.80	Same
Camera-motion compensation	Sparse optical flow	Same
ReID	Enabled; automatic model selection	Same
Additional canonical-ID relinking	Disabled	Enabled
Maximum additional relinking gap	Not applicable	12 processed frames
Additional spatial gate	Not applicable	85(1 + 0.04Δt) pixels
Additional class constraint	Not applicable	Strict majority-class agreement
Minimum counting observations	2	4
Counting line	y = 0.60 H = 432 pixels; above-to-below crossing	Same
Additional event suppression	Disabled	Same class; ≤18 frames and ≤35 pixels

Shared tracker settings were identical between the baseline and improved configurations. Spatial thresholds were expressed in the coordinate system of the 1280 × 720-pixel video frames.

**Table 4:** Detection performance of YOLOv8 and YOLOv8-CBAM under different cultivation environments and cabbage growth stages.

Location/Growth Stage	Configuration	Precision	Recall	mAP@0.50	mAP@0.50:0.95
Overall	YOLOv8	0.85	0.90	0.93	0.58
	YOLOv8-CBAM	0.88	0.92	0.96	0.60
Greenhouse (seedling)	YOLOv8	0.82	0.88	0.90	0.53
	YOLOv8-CBAM	0.88	0.91	0.93	0.59
Greenhouse (cabbage)	YOLOv8	0.84	0.91	0.92	0.55
	YOLOv8-CBAM	0.89	0.89	0.94	0.61
Open field (seedling)	YOLOv8	0.86	0.91	0.93	0.59
	YOLOv8-CBAM	0.89	0.92	0.96	0.62
Open field (cabbage)	YOLOv8	0.90	0.89	0.92	0.58
	YOLOv8-CBAM	0.87	0.93	0.95	0.62

Note: Each detector configuration was trained using the combined greenhouse and open-field training dataset. Environment- and growth-stage-specific results were calculated from the corresponding held-out test subsets using the same trained checkpoint. Overall metrics were calculated from the combined test-set predictions.

**Table 5:** Ablation analysis of attention components in the proposed detector.

Configuration	CA	SA	Run	Precision	Recall	mAP @0.50	mAP @0.5:0.95	Latency (ms)	Detector FPS
YOLOv8	–	–	5	0.85 ± 0.01	0.90 ± 0.008	0.93 ± 0.006	0.58 ± 0.01	15.31	65.3
YOLOv8 + CA	✓	–	5	0.87 ± 0.008	0.89 ± 0.01	0.94 ± 0.005	0.57 ± 0.012	16.58	60.3
YOLOv8 + SA	–	✓	5	0.86 ± 0.009	0.91 ± 0.007	0.95 ± 0.006	0.57 ± 0.011	18.00	55.6
YOLOv8-CBAM	✓	✓	5	0.88 ± 0.007	0.92 ± 0.007	0.96 ± 0.006	0.60 ± 0.008	16.31	61.3

Note: ✓ indicates that the attention component was included; – indicates that it was not included.

**Table 6:** Cabbage counting performance of the six detector–tracker configurations across nine test videos.

Configuration	Mean CE	Mean APE (%)	Mean Counting Accuracy (%)	MAE (Plants)	RMSE (Plants)	R^2^
YOLOv8 + BoT-SORT	−3.00	6.66	93.34	3.00	3.35	0.962
YOLOv8 + Improved BoT-SORT	−2.33	5.23	94.77	2.33	2.65	0.976
YOLOv8-CBAM + Bytetrack	−3.33	5.55	94.45	3.33	3.50	0.959
YOLOv8-CBAM + DeepSORT	−2.22	3.97	96.03	2.22	2.49	0.979
YOLOv8-CBAM + BoT-SORT	−2.22	3.16	96.84	2.22	2.49	0.979
YOLOv8-CBAM + Improved BoT-SORT	−0.56	0.73	99.27	0.56	0.88	0.997

**Table 7:** Comparison of cabbage counting and computational performance among tracking algorithms using the same YOLOv8-CBAM detector across nine test videos.

Tracker	Counting Accuracy (%)	MAE (Plants)	RMSE (Plants)	(R^2^)	Pipeline FPS
ByteTrack	94.45	3.33	3.50	0.959	27.61
DeepSORT	96.03	2.22	2.49	0.979	20.84
BoT-SORT	96.84	2.22	2.49	0.979	23.42
Improved BoT-SORT	99.27	0.56	0.88	0.997	23.38

**Table 8:** Tracking and counting-event performance on four annotated test videos.

Model	MOTA (%)	IDF1 (%)	HOTA (%)	ID Switches	Fragmentations	Missed Plants	Duplicate Counts	False Counts
YOLOv8-CBAM + BoT-SORT	82.2	79.6	74.3	23	8	11	0	0
YOLOv8-CBAM + Improved BoT-SORT	88.4	82.3	83.2	12	3	3	0	0

**Table 9:** Computational performance of the evaluated detector–tracker configurations across the nine test videos.

Configuration	Mean Pipeline FPS
YOLOv8 + BoT-SORT	24.33
YOLOv8 + Improved BoT-SORT	21.14
YOLOv8-CBAM + BoT-SORT	23.42
YOLOv8-CBAM + Improved BoT-SORT	23.38

**Table 10:** Comparison of the present framework with related cabbage detection and counting studies.

Study	Crops/Stage	Data Platform	Detector/Tracker	Detection Metric	Counting Metric
**Chen et al.** [10]	Cabbage seedlings	UAV	TSP-YOLO: improved YOLOv8n with Swin-conv and ParNet attention; DeepSORT-inspired tracking and line-crossing counting	mAP@0.50: 99.4%;	Accuracy 98.02%
**Zhao et al.** [14]	Early-stage cabbage seedlings	UAV	Improved YOLOv11n with ADown, IRCLHead, and LAConv; dynamic NMS and counting logic	mAP@0.50: 99.0%;	Accuracy 99.6%
**Jiang et al.** [25]	Immature and mature field cabbage	Ground-based RGB-D	Improved YOLOv8n with ConvFFN, nonlocal attention, and Swin transformer	mAP@0.50: 93.9%	Not evaluated; study assessed 3D positioning
**Present study**	Seedlings and grown cabbage	RGB	YOLOv8n–CBAM + Improved BoT-SORT	mAP@0.50: 96%	Accuracy 99.27%

## Data Availability

Data presented in this study are contained within the article. Additional data are available from the corresponding author upon reasonable request.

## Appendix A

**Table A1:** Distribution of acquisition groups, images, and annotated plant instances by cultivation environment, growth stage, and dataset partition.

Environment	Growth Stage/Class	Partition	Video/Session	Images (n)	Annotated Instances (n)
Greenhouse	Seedling	Training	5	388	1432
Greenhouse	Grown plant	Training	5	388	1530
Open field	Seedling	Training	5	388	1523
Open field	Grown plant	Training	5	388	1478
Training subtotal	Both classes	Training	20	1552	5963
Greenhouse	Seedling	Validation	3	47	203
Greenhouse	Grown plant	Validation	3	50	189
Open field	Seedling	Validation	3	47	191
Open field	Grown plant	Validation	3	50	202
Validation subtotal	Both classes	Validation	12	194	785
Greenhouse	Seedling	Test	3	47	196
Greenhouse	Grown plant	Test	3	50	178
Open field	Seedling	Test	3	47	195
Open field	Grown plant	Test	3	50	181
Test subtotal	Both classes	Test	12	194	750
Total	Both classes	All partitions		1940	7498

**Table A2:** Acquisition characteristics and manual reference counts of the nine videos used for tracking and counting evaluation.

Video	Site	Date	Growth Stage	Frames	Duration (s)	Manual Count
V1	Open-field	29 October 2024	Cabbage	618	20.60	62
V2	Open-field	29 October 2024	Cabbage	622	20.73	64
V3	Open-field	29 October 2024	Cabbage	620	20.67	62
V4	Open-field	23 July 2024	Seedling	623	20.77	64
V5	Open-field	23 July 2024	Seedling	813	27.10	81
V6	Greenhouse	2 May 2025	Cabbage	800	26.67	77
V7	Greenhouse	2 May 2025	Cabbage	322	10.73	22
V8	Greenhouse	4 February 2025	Seedling	1144	38.13	81
V9	Greenhouse	4 February 2025	Seedling	817	27.23	77

**Table A3:** Per-video cabbage counts obtained using the same YOLOv8-CBAM detector with four tracking configurations.

Tracker	Video	Manual Count	Predicted Count	Signed Error (Plants)	Absolute Error (Plants)	APE (%)
ByteTrack	V1	62	58	−4	4	6.45
ByteTrack	V2	64	59	−5	5	7.81
ByteTrack	V3	62	58	−4	4	6.45
ByteTrack	V4	64	60	−4	4	6.25
ByteTrack	V5	81	79	−2	2	2.47
ByteTrack	V6	77	74	−3	3	3.90
ByteTrack	V7	22	20	−2	2	9.09
ByteTrack	V8	81	77	−4	4	4.94
ByteTrack	V9	77	75	−2	2	2.60
DeepSORT	V1	62	60	−2	2	3.23
DeepSORT	V2	64	62	−2	2	3.13
DeepSORT	V3	62	59	−3	3	4.84
DeepSORT	V4	64	59	−5	5	7.81
DeepSORT	V5	81	79	−2	2	2.47
DeepSORT	V6	77	75	−2	2	2.60
DeepSORT	V7	22	20	−2	2	9.09
DeepSORT	V8	81	80	−1	1	1.23
DeepSORT	V9	77	76	−1	1	1.30
BoT-SORT	V1	62	61	−1	1	1.61
BoT-SORT	V2	64	62	−2	2	3.13
BoT-SORT	V3	62	59	−3	3	4.84
BoT-SORT	V4	64	60	−4	4	6.25
BoT-SORT	V5	81	78	−3	3	3.70
BoT-SORT	V6	77	75	−2	2	2.60
BoT-SORT	V7	22	22	0	0	0.00
BoT-SORT	V8	81	78	−3	3	3.70
BoT-SORT	V9	77	75	−2	2	2.60
Improved BoT-SORT	V1	62	62	0	0	0.00
Improved BoT-SORT	V2	64	63	−1	1	1.56
Improved BoT-SORT	V3	62	62	0	0	0.00
Improved BoT-SORT	V4	64	64	0	0	0.00
Improved BoT-SORT	V5	81	80	−1	1	1.23
Improved BoT-SORT	V6	77	77	0	0	0.00
Improved BoT-SORT	V7	22	22	0	0	0.00
Improved BoT-SORT	V8	81	79	−2	2	2.47
Improved BoT-SORT	V9	77	76	−1	1	1.30

**Table A4:** Computational complexity and model size of YOLOv8n and the attention variants.

Configuration	Parameters (M)	GFLOPs	Model Size (MB)
YOLOv8	3.01	8.2	6.5
YOLOv8 + CA	3.04	8.2	6.8
YOLOv8 + SA	3.03	8.2	6.6
YOLOv8-CBAM	3.08	8.2	6.95
